# Neurometabolic Differences in Psychosis Observed with 7 T Magnetic Resonance Spectroscopy

**DOI:** 10.1093/schbul/sbag137

**Published:** 2026-10-28

**Authors:** Michael-Paul Schallmo, Caroline Demro, Kyle W Killebrew, Aislin A Sheldon, Cheryl A Olman, Scott R Sponheim, Małgorzata Marjańska

**Affiliations:** Department of Psychiatry and Behavioral Sciences, University of Minnesota, Minneapolis, MN 55454, United States; Department of Psychiatry and Behavioral Sciences, University of Minnesota, Minneapolis, MN 55454, United States; Department of Psychology, University of Minnesota, Minneapolis, MN 55455, United States; Department of Psychiatry and Behavioral Sciences, University of Minnesota, Minneapolis, MN 55454, United States; Department of Psychiatry and Behavioral Sciences, University of Minnesota, Minneapolis, MN 55454, United States; Department of Psychology, University of Minnesota, Minneapolis, MN 55455, United States; Center for Magnetic Resonance Research, Department of Radiology, University of Minnesota, Minneapolis, MN 55455, United States; Department of Psychiatry and Behavioral Sciences, University of Minnesota, Minneapolis, MN 55454, United States; Veterans Affairs Medical Center, Minneapolis, MN 55417, United States; Center for Magnetic Resonance Research, Department of Radiology, University of Minnesota, Minneapolis, MN 55455, United States

**Keywords:** magnetic resonance spectroscopy, schizophrenia, bipolar disorder, human connectome project, *N*-acetyl-aspartate, glucose

## Abstract

**Background:**

Altered brain chemistry is thought to contribute to impaired cognition and perceptual functioning in people with psychotic psychopathology (PwPP). As heritable genetic factors shape the development of psychosis, alterations in brain chemistry may extend to biological relatives of PwPP. Magnetic resonance spectroscopy (MRS) is a non-invasive method for quantifying concentrations of various neurochemicals in the human brain. Previous studies using MRS have generally focused on specific diagnoses (eg, schizophrenia), rather than PwPP trans-diagnostically, and comparably few studies have examined relatives. The goal of the current study was to assess neurometabolic differences in PwPP and relatives using MRS in a large number of participants.

**Study Design:**

As part of the Psychosis Human Connectome Project, we acquired ultra-short echo time (8 ms) MRS data at 7 tesla in both occipital and prefrontal cortices from 64 PwPP, 42 first-degree biological relatives, and 43 controls.

**Study Results:**

We observed lower levels of *N*-acetyl-aspartate (NAA) in the occipital lobe in PwPP and relatives versus controls, and a trend toward lower *N*-acetyl-aspartylglutamate in prefrontal cortex in PwPP versus controls. Lower OCC NAA levels were also evident in diagnostic subgroups of PwPP (schizophrenia and bipolar disorder) compared to controls. We also observed higher levels of glucose in both occipital and prefrontal cortices in PwPP. Hierarchical clustering analyses showed that higher glucose levels were linked to higher psychiatric symptoms, poorer cognition, and impaired visual task performance.

**Conclusions:**

Together, our findings point to alterations in neural metabolism across multiple brain areas in PwPP that are associated with impaired cognitive and perceptual functioning.

## Introduction

Altered brain chemistry in people with psychotic psychopathology (PwPP) is a prominent hypothesis, including altered glutamatergic neurotransmission,^1^^,^^2^ and impaired functioning of the inhibitory neurotransmitter γ-aminobutyric acid (GABA).^3–5^ For example, pharmacological and genetic data suggest there may be reduced excitation via glutamatergic *N*-methyl-D-aspartate receptors on inhibitory interneurons in prefrontal cortex (PFC), which could alter the balance of excitation and inhibition.^6^^,^^7^ Psychotic disorders may also involve broader neurochemical changes related to brain metabolism.^8^^,^^9^ Magnetic resonance spectroscopy (MRS) is a non-invasive method for assessing brain chemistry. A number of MRS studies have been conducted in psychotic disorders; many have focused on schizophrenia,^10–18^ with fewer having compared across diagnoses, or considered PwPP trans-diagnostically,^18–20^ and published findings have varied. Metabolites that have shown more consistent differences (typically lower concentrations) in PwPP compared to controls include glutamate and GABA, as well as glutamine (involved in glutamate cycling), glutathione (an antioxidant), *N*-acetyl-aspartate (NAA; a marker of neuronal health and metabolic function), and *N*-acetyl-aspartyl-glutamate (NAAG; a glutamatergic peptide neurotransmitter). A smaller number of MRS studies have been conducted among biological relatives of PwPP^21–27^ and suggest brain metabolite levels in relatives may fall on a continuum between controls and PwPP.

Disruptions in perception and cognition are common and significant among PwPP; they are often not addressed by antipsychotics, which can hinder recovery and real-world functioning.^28–30^ Although altered visual perception among PwPP is well documented,^31–35^ the neural bases of visual dysfunction remain unclear. Differences in brain chemistry may contribute to visual dysfunction among PwPP, but this link has not yet been firmly established.^12^^,^^36^^,^^37^

The goals of the current study were: (1) to use state-of-the-art methods to quantify brain metabolite levels in a large group of PwPP using 7 tesla (T) MRS, and (2) to assess how brain metabolites were associated with measures of visual, cognitive, and psychiatric functioning. Acquiring the data at 7 T provided us with higher SNR and improved the ability to study specific metabolites of interest (ie, separately quantifying glutamate and glutamine, higher reliability for measuring GABA without spectral editing), as compared to similar 3 T methods.^38–42^ To date, relatively few studies have used 7 T MRS to examine brain chemistry in PwPP.^20^^,^^24^^,^^26^^,^^43^^,^^44^ Novel aspects of our work include: (1) examining associations between measures of brain chemistry from 7 T MRS and visual function from neuroscience-informed tasks,^45^ (2) studying PwPP transdiagnostically, as well as first-degree biological relatives, and (3) collecting and making our data publicly available as part of the Psychosis Human Connectome Project,^45^^,^^46^ to facilitate future studies examining links between brain metabolite levels and other imaging measures (ie, from structural MRI, diffusion MRI, resting state and/or task functional MRI).

Because perceptual abnormalities are a defining characteristic of psychosis, we chose a mid-occipital (OCC) volume of interest (VOI) that included primary visual cortex, which plays a key role in perception of contrast, orientation, and motion.^47^ As we were also interested in higher-level cognitive impairments in PwPP, we chose a second VOI within the dorsomedial PFC, a region involved in decision making and cognitive control.^48–50^ In our first set of analyses, we took a transdiagnostic approach to studying psychosis. We also examined first-degree biological relatives of PwPP, who on average share 50% of their genes with someone with psychosis. This allowed us to investigate whether genetic liability for psychosis is associated with differences in brain chemistry. Second, we compared people with schizophrenia, bipolar disorder with psychotic features, and controls, to better understand the relevance of our findings to specific diagnostic categories. We hypothesized that impaired visual and cognitive functioning would be associated with lower glutamatergic (ie, glutamate, glutamine, NAAG) or GABA levels in PwPP vs. controls, reflecting excitatory and/or inhibitory differences.^1–7^^,^^10–12^^,^^14–17^^,^^19^^,^^20^^,^^51^ Our alternative hypotheses were that such impairments would be associated with either lower glutathione levels (reflecting impaired antioxidant function), and/or lower NAA levels (reflecting metabolic differences) in PwPP.^18^^,^^20^^,^^52^

## Methods

Our experiments were conducted as part of the Psychosis Human Connectome Project, and our methods are fully described in our recent publications.^45^^,^^46^ Essential methodological information for the current study is provided below. Additional details are provided in Supplementary material.

### Participants

We collected MRS data from 3 groups (age: 18-59 years): control participants with no family history of psychosis (controls, *n* = 44), first-degree biological relatives of individuals with psychosis (relatives, *n* = 44), and people with a history of psychotic psychopathology (PwPP, *n* = 66). Of these, 5 individuals (1 control, 2 relatives, 2 PwPP) were excluded after participation (eg, for having a visual abnormality) and were not considered further. Our final sample included 43 controls, 42 relatives, and 64 PwPP. Demographic information is presented in Table 1.

**Table 1:** Participant Demographics

	Controls, *n* = 43	Relatives, *n* = 42	PwPP, *n* = 64	Statistics
Age (years)^a^	37.8 (12.2)	43.7 (12.8)	38.1 (11.6)	***F***_**2,146**_ **= 3.4,** ***P* = .037**
Sex assigned at birth	23F, 20M	28F, 14M	30F, 34M	*Χ* ^**2**^_2, n = 149_ = 4.0, *P* = .13
Education (years)^b^	16.2 (2.1)	15.6 (2.1)	14.2 (2.3)	***F***_**2,146**_ **= 11.2,** ***P* = 3 × 10**^**−5**^
BMI^c^	25.3 (4.91)	28.9 (6.07)	29.3 (5.77)	***×*** ^**2**^_**2, n = 129**_ **= 12.2,** ***P* = .002**
Visual acuity (logMAR)	0.04 (0.13)	0.04 (0.13)	0.06 (0.17)	*F*_2,99_ = 0.36, *P* = .7
Mars letter contrast sensitivity^d^	1.84 (0.06)	1.81 (0.05)	1.79 (0.07)	***F***_**2,145**_ **= 6.8,** ***P* = .002**
Estimated IQ^e^	108 (9.3)	104 (10.0)	98 (10.7)	***F***_**2,146**_ **= 14.4,** ***P* = 2 × 10**^−**6**^
BACS^f^	0.37 (0.54)	0.02 (0.73)	−0.65 (0.69)	***F***_**2,145**_ **= 32.5,** ***P* = 2 × 10**^**−12**^
BPRS^g^	26.7 (3.4)	30.9 (6.9)	42.0 (10.5)	***×*** ^**2**^_**2, n = 125**_ **= 56.0,** ***P* = 7 × 10**^**−13**^
SGI^h^	35.5 (19.6)	51.9 (31.5)	69.7 (28.4)	***Χ*** ^**2**^_**2, n = 149**_ **= 35.5,** ***P* = 2 × 10**^**−8**^
SPQ^i^	8.1 (8.2)	15.2 (13.5)	30.5 (14.5)	***Χ*** ^**2**^_**2, n = 149**_ **= 58.0,** ***P* = 3 × 10**^**−13**^
PID-5 psychoticism^j^	0.27 (0.28)	0.40 (0.41)	1.17 (0.62)	***Χ*** ^**2**^_**2, n = 145**_ **= 64.9,** ***P* = 8 × 10**^**−15**^
SAPS	-	-	4.80 (4.30)	-
SANS	-	-	6.10 (3.95)	-
CPZ equivalents (mg/day)	-	0.02 (0.15)	18.2 (125)	-
Duration of psychotic illness (years)	-	-	15.8 (10.0)	-
Diagnoses of participants [or of related probands]				
Schizophrenia	0	0 [21]	35	-
Schizoaffective	0	0 [8]	9	-
Bipolar disorder with psychotic features	0	0 [13]	20	-
Other (eg, MDD, generalized anxiety, cannabis dependence)	1	21	0	-
None	42	21	0	-

Data are mean (SD) unless otherwise noted. The statistics column shows the test statistic value and *P-*value for differences across all 3 groups (bold: *P* < .05). Superscript letters denote significant *post-hoc* differences between groups at *P* < .05, as noted below. Visual acuity is reported in logMAR (eg, 0 indicates 20/20). For relatives, under Diagnoses, the number of related individuals with a particular psychotic disorder diagnosis is listed in square brackets. Abbreviations: BMI = body mass index; CPZ = chlorpromazine dose equivalents^53^^,^^54^; MDD = Major Depressive Disorder; OCC = Occipital cortex; PFC = Prefrontal cortex.

^a^Relatives > controls, relatives > PwPP; *F*-values >4.74, *P*-values <.032. Note that participant age range was similar across groups; controls: 18-59, relatives: 19-59, PwPP: 18-59.

^b^Controls > PwPP, relatives > PwPP; *F*-values >9.03, *P*-values <.003.

^c^Relatives > controls, PwPP > controls; *Χ*^2^ values >6.51, *P*-values <.011.

^d^Controls > relatives, controls > PwPP; *F*-values >5.74, *P*-values <.019.

^e^Controls > PwPP, relatives > PwPP; *F*-values >9.37, *P*-values <.003.

^f^Controls > relatives, controls > PwPP, relatives > PwPP; *F*-values >6.41, *P*-values <.013.

^g^Relatives > controls, PwPP > controls, PwPP > relatives; *Χ*^2^ values >9.47, *p* values <0.002

^h^Controls > relatives, controls > PwPP, relatives > PwPP; *Χ*^2^ values >6.65, *P*-values <.010.

^i^Relatives > controls, PwPP > controls, PwPP > relatives; *Χ*^2^ values >6.46, *P*-values <.011.

^j^PwPP > controls, PwPP > relatives; *Χ*^2^ values >35.8, *P*-values <2 × 10^−9^.

Participants in the PwPP group were stable outpatients with a history of schizophrenia, schizoaffective disorder, or bipolar I disorder with psychotic features. Research diagnoses were made using Structured Clinical Interview for DSM-IV-TR^55^ by postdoctoral associates and trained research staff at a separate research visit prior to scanning, generally a few months before the MRS visit. Relatives had a biological parent, sibling, or child with a history of a psychotic disorder (confirmed by diagnostic interview), but not all probands participated in MRS. Relatives included people with and without current psychiatric diagnoses (Table 1), but none with psychosis. Controls had no personal or immediate family history of psychosis spectrum disorders. One control had a diagnosis of generalized anxiety; excluding this individual did not affect our results (data not shown). There were 21 pairs of related participants: 18 PwPP-relative pairs, and 3 relative-relative pairs (for whom a related PwPP did not participate). 46 PwPP and 1 relative were taking antipsychotics at the time of scanning; 25 PwPP and 3 relatives were taking benzodiazepines; 22 PwPP and 2 relatives were taking mood stabilizers. All participants provided written informed consent prior to participation, and were compensated $20 per hour. Our procedures complied with the Declaration of Helsinki and were approved by the Institutional Review Board at the University of Minnesota.

### MR Scanner

We acquired 7 T MRS data using a Siemens MAGNETOM scanner equipped with an 8-kW RF power amplifier and body gradients with 70 mT/m maximum amplitude and 200 T/m/s maximum slew rate. We used a custom-built radio frequency (RF) head coil with 2 surface ^1^H quadrature transceivers, one for OCC and one for PFC.

### Experimental Protocol

#### MR Spectroscopy

The following information conforms to established MRS reporting standards.^45^^,^^56^ We used a stimulated echo acquisition mode (STEAM) sequence with 3D outer volume suppression interleaved with variable power and optimized relaxation delay (VAPOR) water suppression^57^ with repetition time (TR) = 5 s, echo time (TE) = 8 ms, mixing time (TM) = 32 ms, number of complex data points = 2048, spectral bandwidth = 6000 Hz. We chose an ultra-short TE to mitigate the potential confound of shorter *T_2_* relaxation times in PwPP.^52^^,^^58–61^ For each VOI, we acquired 96 shots with a scan duration of 8 min. We also acquired a water reference scan for each VOI (1 average, transmitter frequency = 4.7 parts per million [ppm], no water suppression) for quantifying metabolite concentrations relative to water, and to facilitate eddy current compensation. We used FAST(EST)MAP^62^^,^^63^ to perform shimming within each VOI in order to obtain a water linewidth ≤15 Hz. We acquired MRS data from participants at rest.

The OCC VOI size was 30 mm (left–right) × 18 mm (anterior–posterior) × 18 mm (inferior–superior). We positioned the VOI within the medial occipital lobe, superior to the cerebellar tentorium, anterior to the occipital pole, and posterior to the occipitoparietal junction (Figure 1). This VOI encompasses regions including primary visual cortex that are known to play a role in fundamental aspects of visual perception.

**Figure 1:**
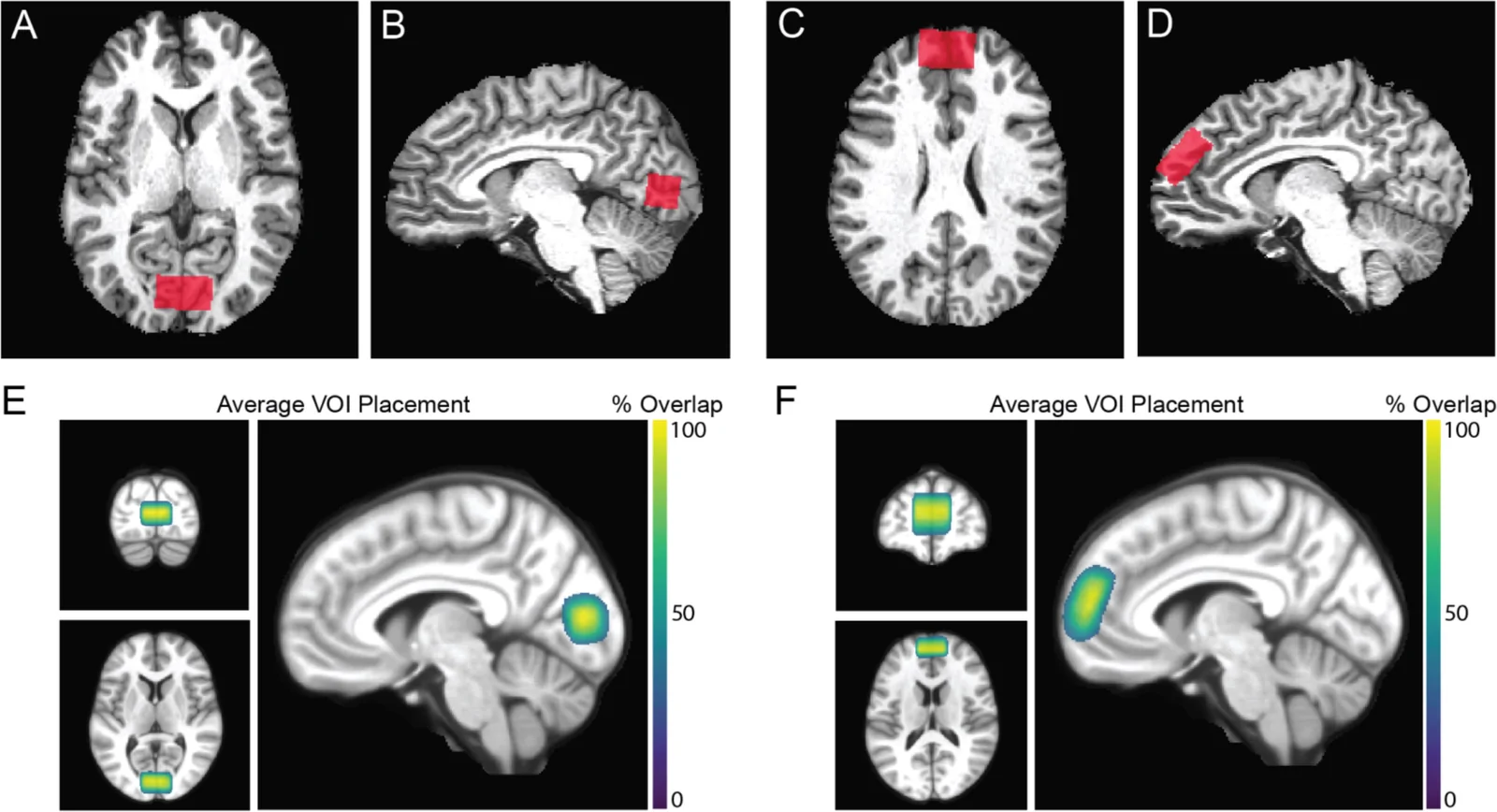
Occipital and prefrontal volumes of interest (VOIs). (A) Axial and (B) sagittal images from an individual participant showing position of the occipital VOI. Panels (C) and (D) show the same, but for the prefrontal VOI. (E) Average occipital VOI placement across data sets. Color bar shows % overlap across data sets. Voxels with ≥ 30% overlap are shown. VOIs were transformed from individual to MNI space. (F) Same, but for the prefrontal VOI. Figures adapted from Schallmo et al.^45^

The PFC VOI size was 30 mm (left–right) × 30 mm (anterior–posterior) × 15 mm (inferior–superior). This VOI was obliquely oriented within the dorsomedial PFC, superior to the anterior cingulate, along a line extending 135° from the anterior edge of the genu of the corpus callosum (Figure 1). Total time in the MR scanner for both VOIs combined, including setup and shimming, was approximately 80 min.

#### Visual Tasks

We obtained task-based measures of visual contrast surround suppression (CSS), contour object perception (COP), and bi-stable perception of structure-from-motion (SFM) outside the scanner, prior to MRS data collection. These tasks were informed by studies linking perception and neural function in healthy adults and animal models.^45^ Previous work suggests altered perception in these domains among PwPP,^64–66^ which may be linked to differences in neurometabolites. CSS involved judging which of 2 grating stimuli was higher contrast, in the presence or absence of suppressive surrounding stimuli, with difficulty adjusted across trials via an adaptive staircase procedure. COP involved judging the orientation of an egg-shaped contour, with contour elements embedded in a field of distractors, again using a staircase procedure. SFM examined the rate of bi-stable switching of perceived motion direction in the rotating cylinder illusion.

#### Clinical Measures

We assessed mental health symptom severity, personality traits, and perceptual responsiveness outside the scanner prior to MRS data collection.^45^^,^^46^ This included the Brief Psychiatric Rating Scale (BPRS),^67^ Scale for the Assessment of Negative Symptoms,^68^ Scale for the Assessment of Positive Symptoms,^69^ and visual acuity,^70^ assessed on the day of MRS scanning. Other measures collected in a separate session (typically a few months before 7 T scanning) included the Brief Assessment of Cognition in Schizophrenia (BACS; Z-scored),^71^ Sensory Gating Inventory (SGI),^72^ Schizotypal Personality Questionnaire (SPQ),^73^ Personality Inventory for the DSM-5 (PID-5; psychoticism factor score),^74^ and Mars Letter Contrast Sensitivity.^75^ Estimated IQ was assessed using the Wechsler Adult Intelligence Scale.^76^

### Data Processing and Quality Assessment

MRS data processing included eddy current compensation for metabolite and water reference spectra. We then performed frequency and phase correction, as previously described.^45^

We quantified metabolite concentrations from 0.5 to 4.1 ppm using LCModel version 6.3-1N.^77^  Figure 2 shows example spectra from OCC and PFC of individual participants. We used a basis set obtained from a previous study,^78^ which included 18 metabolites and experimentally-measured macromolecules. Spectra were fitted using a stiff baseline (spline constraint parameter DKNTMN = 5) without soft constraints on metabolite concentrations, to avoid possible quantification inaccuracies.^79–81^ We scaled our metabolite concentrations (millimolar) relative to unsuppressed water. We applied corrections for gray matter (GM), white matter (WM), and cerebrospinal fluid (CSF) content (ie, water content, *T_1_* and *T_2_* relaxation times) within each VOI in each participant (Supplementary Table 1). The tissue fraction within each VOI in each participant was quantified using individual GM and WM segmentations from FreeSurfer (v5.3.0^82^). Since glucose is present in the CSF^83^ and its concentration in the CSF varies,^84^ we have reported glucose concentrations within the VOI rather than the brain tissue (unlike other metabolites), as we did not want to make assumptions about the glucose concentration within the CSF.

**Figure 2:**
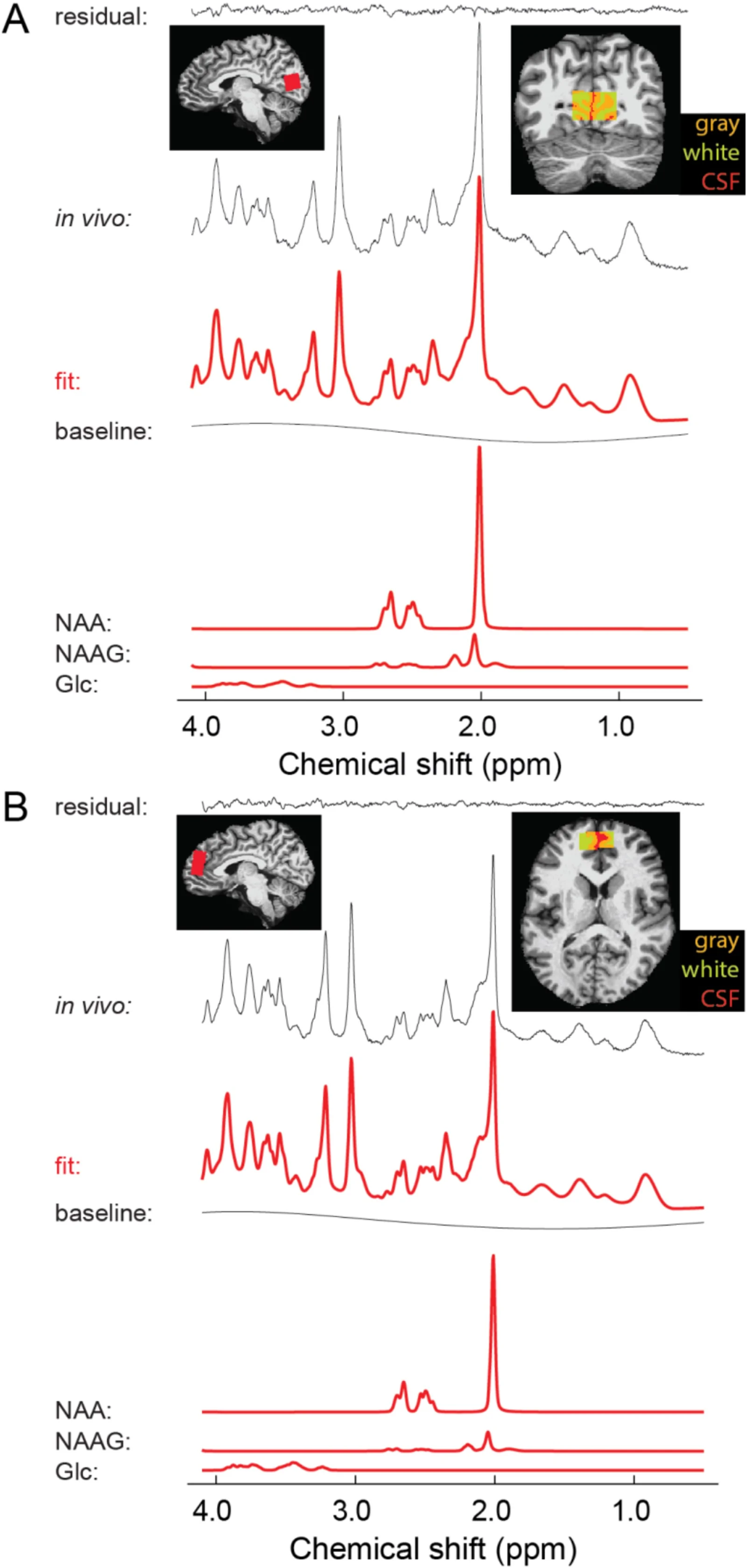
Example MR spectra. (A) OCC spectrum from an individual participant (black) plus LCModel fit (red) and residual. The bottom rows show NAA, NAAG, and glucose (Glc) signals, as fitted by LCModel. Insets show OCC VOI position, top left: sagittal, with VOI in red; top right: coronal, with GM (orange), WM (yellow), and CSF (red) highlighted. (B) Same, but for the PFC VOI; top right inset shows axial view.

We assessed data quality for OCC and PFC VOIs separately using the following metrics: H_2_O linewidth, LCModel linewidth, LCModel signal-to-noise ratio. Overall, data quality was high and similar across groups (Supplementary Table 2). In total, 5 OCC data sets (2 relatives, 3 PwPP) and 9 PFC data sets (1 control, 5 relatives, 3 PwPP) were excluded due to poor quality. Cramér-Rao Lower Bounds in absolute units are reported in Supplementary Table 6, but were not used for data quality thresholds.^85^

### Data Analysis and Statistics

We performed our analyses using MATLAB. We conducted 2 sets of analyses comparing metabolite concentrations from each VOI between groups. Our first analysis focused on 6 metabolites that were chosen a priori: GABA, glutamate (Glu), glutamine (Gln), glutathione (GSH), NAA, and NAAG. We chose these 6 metabolites based on a review of the literature^10^^,^^14^^,^^16–20^^,^^86–88^ prior to data analysis, which suggested that concentrations of these metabolites in OCC and/or PFC may be altered in PwPP. As a number of our metabolite concentrations were not normally distributed, and variance was not always equivalent across groups, we used Kruskal–Wallis 1-way non-parametric ANOVAs to assess group differences. Because we considered the group comparisons for each of these 6 metabolites as separate hypotheses defined a priori based on the literature, we did not perform corrections for multiple comparisons in this case. We also carried out a second set of exploratory group comparisons for 10 additional metabolites: ascorbic acid (Asc), aspartic acid (Asp), total choline (tCho), total creatine (tCr), glucose (Glc), *myo*-inositol (mIns), phosphorylethanolamine (PE), *scyllo*-inositol (sIns), taurine (Tau), and macromolecules (MacM). We did not examine group differences for lactate or lipids, as they were not fitted consistently across all participants. We used summed values for total choline and total creatine, as the fit values for glycerophosphorylcholine and phosphorylcholine were highly correlated, as were creatine and phosphocreatine. We applied statistically conservative false discovery rate (FDR) corrections for multiple comparisons. We also performed follow up analyses to examine the influence of covariates.

We performed hierarchical clustering analyses^89^ to examine multivariate relationships between metabolite concentrations from MRS, visual task performance metrics, and clinical measures in a data driven manner. This involved computing Spearman’s correlation values across all pairwise combinations of 30 variables (16 OCC metabolites, plus 6 visual task metrics, and 8 clinical measures; referred to as OCC clustering hereafter; Supplementary Table 3) or 46 variables (the 30 above, plus 16 PFC metabolites; OCC + PFC clustering hereafter; Supplementary Table 4) across all participants. We used principal components analysis to determine the appropriate number of clusters (Supplementary Figure 12). We examined the statistical significance of our clustering results using permutation analyses.

### Data and Code Availability

Our data are publicly available through the National Institute of Mental Health Data Archive (nda.nih.gov/edit_collection.html?id=3162). Our data processing code is available from GitHub (github.com/mpschallmo/PsychosisHCP).

## Results

### Brain Metabolite Levels

We compared concentrations across 3 groups (controls, relatives, PwPP) for the following 6 metabolites of interest, chosen a priori: GABA, glutamate, glutamine, glutathione, NAA, and NAAG.

In OCC, initial non-parametric tests showed NAA levels differed significantly across groups (Kruskal-Wallis, *Χ*^2^_2, n = 141_ = 7.46, *P* = .024) and were lower for both PwPP and relatives compared to controls (post-hoc Kruskal–Wallis tests, *Χ*^2^ values >4.35, *P*-values <.037; Figure 3A). Groups differed significantly in terms of age and BMI, and there were twice as many females as males among relatives, whereas controls and PwPP were more evenly distributed (Table 1). To address this, we performed follow-up analyses with age, sex assigned at birth, and BMI included as covariates in linear mixed-effects models. The difference in NAA between controls and PwPP (but not relatives) remained significant with these covariates. Non-parametric analyses also showed a trend toward a difference in OCC NAAG levels across groups (*Χ*^2^_2, n = 141_ = 5.30, *P* = .071; Supplementary Figure 2K), but this was not significant with covariates included. Age was significantly associated with NAA (parameter estimate [SE] = −0.014 [0.004], *t*_156_ = -3.38, *P* = 9 × 10^-4^) and NAAG in OCC (parameter estimate [SE] = 0.006 [0.001], *t*_156_ = 3.51, *P* = 6 × 10^-4^). OCC NAAG also correlated with years of illness among PwPP (Spearman’s *r*_45_ = 0.32, *P* = .027). We saw non-significant trends toward lower OCC glutamate in PwPP vs. both controls and relatives with covariates included (but not without). Other a priori group comparisons for metabolites in OCC were not significant (see Supplementary Figure 2, Supplementary Tables 5 and 7).

**Figure 3:**
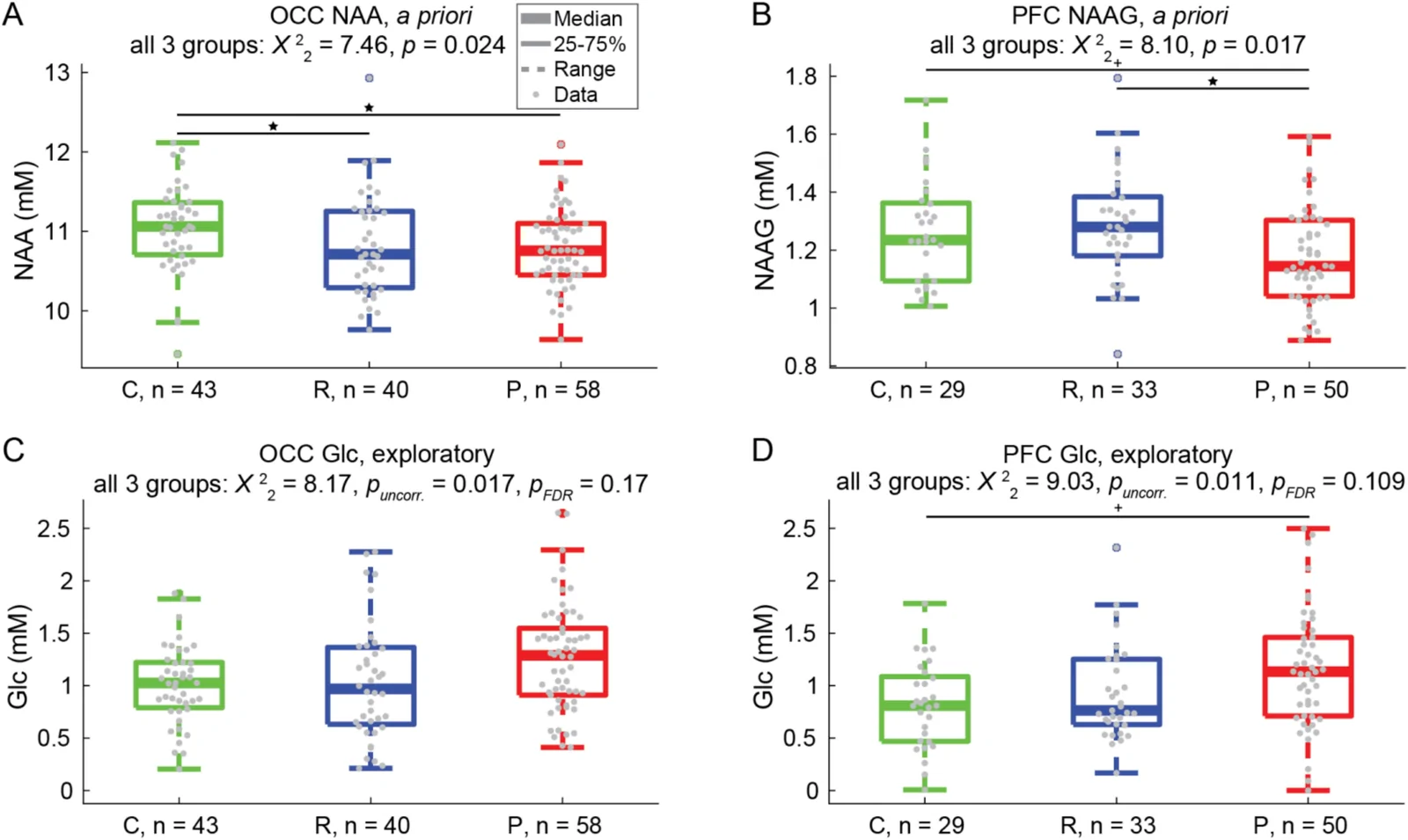
MRS results. We observed significant group differences in (A) NAA in OCC and (B) NAAG in PFC. Differences were also observed in glucose (Glc) in both OCC and PFC (C & D, respectively). C = controls, R = relatives, P = people with psychotic psychopathology. Thick lines show group medians, boxes show 25%-75%, whiskers show 1.5× interquartile range, dots show individual data points. Stars and lines indicate significant differences between 2 groups at *P* < .05, plus at *P* < .1.

In PFC, initial non-parametric tests showed a significant group difference in NAAG levels (*Χ*^2^_2, n = 112_ = 8.10, *P* = .017), which tended to be lower in PwPP compared to both relatives (*Χ*^2^_1, n = 83_ = 7.16, *P* = .008) and controls (a trend-level difference, *Χ*^2^_1, n = 79_ = 3.57, *P* = .059; Figure 3B). However, group differences in PFC NAAG were no longer significant with covariates in linear mixed effects models. NAAG in PFC varied significantly with age across groups (parameter estimate [SE] = 0.006 [0.001], *t*_113_ = 4.43, *P* = 2 × 10^−5^) and with years of illness among PwPP (Spearman’s *r*_40_ = 0.35, *P* = .024). When including covariates (but not without), we saw non-significant trends toward lower GABA, glutathione, and NAA in PFC for PwPP vs. controls. Other a priori group comparisons for metabolites in PFC were not significant (Supplementary Figure 3, Supplementary Tables 5 and 7).

We performed exploratory group comparisons for 10 additional metabolites in both OCC and PFC, with FDR correction for multiple comparisons. Of these, only glucose showed notable group differences. In PFC, there was a trend toward a group difference (*Χ*^2^_2, n = 112_ = 9.03, *P_uncorr._* = .011, *P_FDR_* = .109), with higher glucose levels in PwPP versus controls (*Χ*^2^_1, n = 79_ = 7.60, *P_uncorr._* = .006, *P_FDR_* = .059; Figure 3D), and relatives showing intermediate values (relatives vs. PwPP: *Χ*^2^_1, n = 83_ = 4.62, *P_uncorr._* = .032, *P_FDR_* = .3). A similar pattern was observed in OCC, but was not significant after correcting for multiple comparisons (difference across all 3 groups: *Χ*^2^_2, n = 141_ = 8.17, *P_uncorr._* = .017, *P_FDR_* = .17; controls vs. PwPP: *Χ*^2^_2, n = 141_ = 7.99, *P_uncorr._* = .012, *P_FDR_* = .104; Figure 3C). BMI correlated significantly with glucose in OCC (Spearman’s *r*_120_ = 0.26, *P* = .004), but not PFC (*r*_92_ = 0.13, *P* = .2; Supplementary Figure 4). Follow-up analyses suggested that age, sex, CSF fraction, and tissue composition within the VOI may not have been sufficient to account for the observed group differences in glucose (see Supplementary Table 9; Supplementary Figure 4). For data on other metabolites, see Supplementary Figures 2 and 3, Supplementary Table 5.

In PwPP, metabolite levels (6 chosen a priori, plus glucose) did not vary significantly with antipsychotic medication dose (*P*-values >.3; Supplementary Tables 8 and 9).

To understand the relevance of our findings with respect to specific diagnostic categories, we compared metabolite levels between controls, a sub-group of PwPP with diagnoses of schizophrenia (*n* = 29), and those with bipolar disorder with psychotic features (*n* = 20). People with schizoaffective disorder (n = 9) were excluded, due to low sample size. In OCC, group differences in NAA were again significant (*Χ*^2^_2, n = 91_ = 6.50, *P* = .039; Supplementary Figure 5L). OCC NAA was lower in bipolar disorder vs. controls (*Χ*^2^_1, n = 62_ = 5.31, *P* = .021), with a trend in the same direction for schizophrenia vs. controls (*Χ*^2^_1, n = 71_ = 3.19, *P* = .074). We also saw significant differences in *myo*-inositol in OCC (*Χ*^2^_2, n = 91_ = 12.6, *P_uncorr._* = .002, *P_FDR_* = .018; Supplementary Figure 5K); people with bipolar disorder had lower *myo*-inositol in OCC than controls (*Χ*^2^_1, n = 63_ = 11.5, *P_uncorr._* = 7 × 10^-4^, *P_FDR_* = .007) and people with schizophrenia (*Χ*^2^_1, n = 49_ = 7.99, *P_uncorr._* = .005, *P_FDR_* = .047). Differences for other metabolites in OCC and PFC were not significant (Supplementary Figures 5 and 6, Supplementary Table 10).

### Associations with Visual Task and Clinical Measures

Because our initial hypotheses focused on altered excitation and inhibition in PwPP, we examined bi-variate correlations of glutamate and GABA levels with visual task and clinical measures (14 variables; Supplementary Table 3). For glutamate and GABA in OCC, correlations were not significant across all participants, nor PwPP alone (*P_uncorr._* values >.06). Likewise, correlations with glutamate and GABA in PFC did not survive correction for multiple comparisons (*P_FDR._* values >.4). To further explore relationships between brain metabolites and our visual and psychiatric measures using a method that is less limited by multiple comparisons, we turned to a data driven (hierarchical clustering) approach.

Our OCC clustering analysis showed that glucose in OCC (red text in Figure 4) was part of a cluster of variables that included psychiatric symptom and cognitive measures (eg, Brief Psychiatric Rating Scale total score, Brief Assessment of Cognition in Schizophrenia), as well as measures of visual task performance (ie, CSS contrast discrimination thresholds, SFM bi-stable illusion switch rates). Other MRS measures clustered with one another (eg, green and cyan dashed boxes in Figure 4) and not with the visual and clinical measures. We performed permutation analyses to assess the statistical significance of our hierarchical clustering results. For the cluster that included glucose in OCC (red dashed box in Figure 4), the average correlation (*r* value) between variables within the cluster was significantly higher than for shuffled data (permutation test with data shuffled across participants: *P* < .001; with variables shuffled across clusters: *P* = .020).

**Figure 4:**
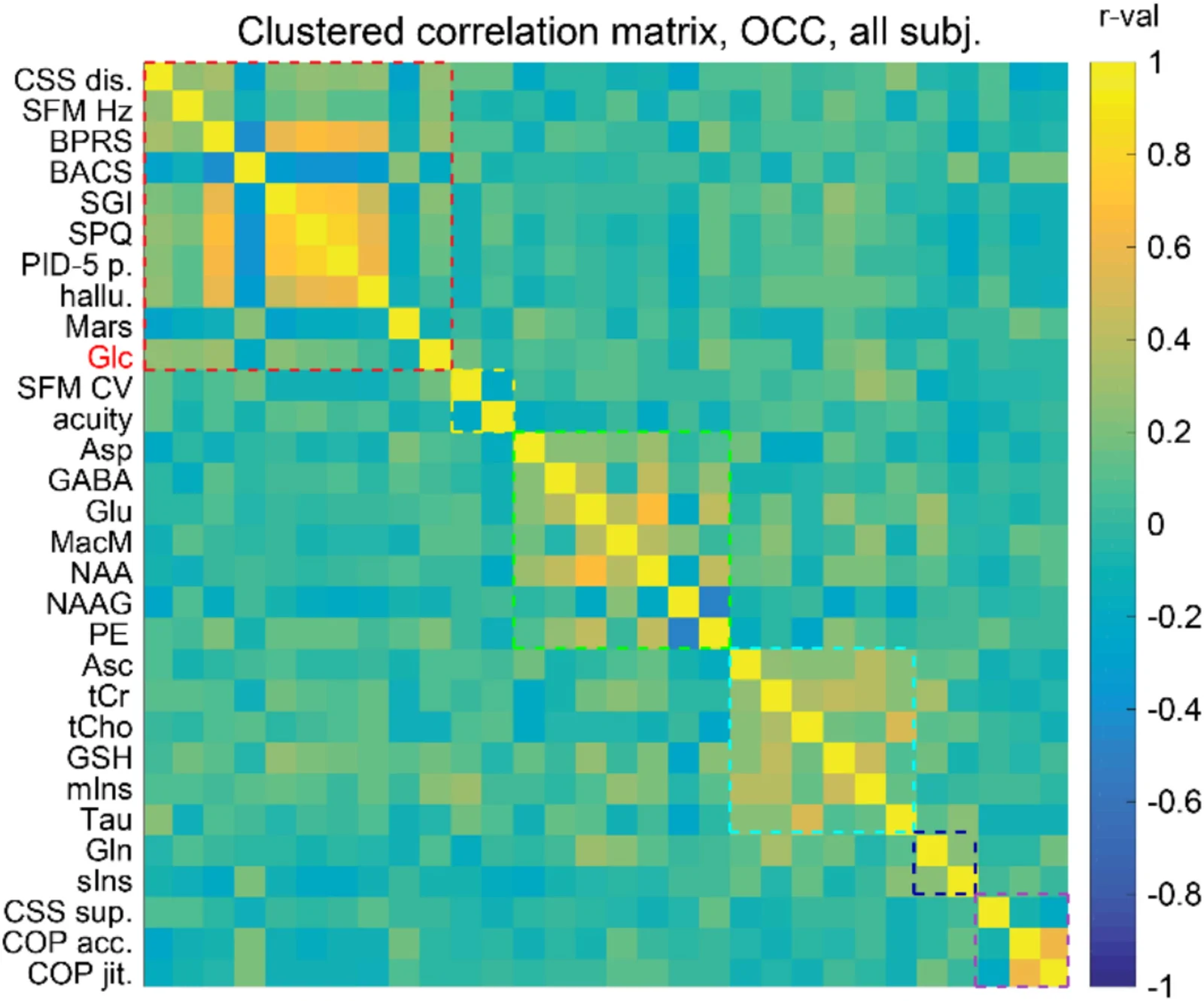
OCC hierarchical clustering results. Cells show pairwise Spearman correlation *r* values for measures from visual tasks (CSS discrimination and suppression, COP accuracy and jitter threshold, SFM switch rate [Hz] and coefficient of variance [CV]), clinical assessments (BPRS, BACS, SGI, SPQ, PID-5 psychoticism, hallucinations, visual acuity, Mars contrast sensitivity), and MRS in OCC (Asc, Asp, tCr, GABA, Glc, Gln, Glu, tCho, GSH, mIns, MacM, NAA, NAAG, PE, sIns, Tau). Colored dashed lines indicate the clusters. Red text indicates glucose, which was the only MRS variable that tended to cluster with visual task and clinical measures (red dashed box).

We saw similar results when we repeated our clustering analysis using OCC data from PwPP only (Supplementary Figures 8 and 9), suggesting that the covariance structure observed in PwPP alone is similar to that across all groups. We also saw similar results for OCC + PFC clustering across all participants (Supplementary Figures 10 and 11). Together, the results of our hierarchical clustering analyses indicate that across participants, higher glucose levels in OCC and PFC are associated with higher psychiatric symptom levels, poorer cognition, and impaired visual task performance.

## Discussion

Using 7 T MRS data we collected as part of the Psychosis HCP, we examined differences in the concentrations of 16 neurochemicals between PwPP, biological relatives, and controls in both OCC and PFC. Our most novel finding came from our exploratory analyses, which revealed higher glucose levels in PwPP in PFC (at a trend level, after statistically conservative FDR correction), and a similar pattern in OCC (which did not survive correction). Hierarchical clustering analyses indicated that glucose levels were associated with measures of psychiatric and visual functioning. Among the 6 metabolites we examined a priori, our most robust finding was lower NAA levels in OCC in PwPP versus controls. Together, our results suggest a pattern of metabolic alterations across brain regions in PwPP that is linked to visual and cognitive impairment, and psychiatric symptom severity.

Our results suggested higher glucose levels in PwPP in PFC, and to a lesser extent in OCC. Hierarchical clustering indicated glucose levels in OCC and PFC were part of a group of associated variables that also included NAAG, as well as measures of psychiatric symptoms, cognition, and visual task performance. The association between these variables may suggest a single latent construct underlying aspects of cognitive and perceptual dysfunction in people with psychosis, such that PwPP who have higher concentrations of brain glucose also experience greater cognitive and perceptual impairments. We have recently published studies from the Psychosis Human Connectome Project showing differences in visual contrast discrimination and surround suppression,^64^ as well as contour object perception^66^ among PwPP (but not relatives) vs. controls, whereas both PwPP and relatives showed faster bi-stable switching during structure-from-motion.^65^ Our current results extend these findings, and suggest that higher brain glucose levels may be linked to visual dysfunction among PwPP.

Impaired brain metabolism among PwPP has also been found in studies using other imaging modalities, including reports of lower glucose metabolism as observed by ^18^FDG-PET, especially in the frontal lobe in people with schizophrenia.^9^ Higher glucose levels in PFC, as suggested by our ^1^H-MRS results, appears broadly consistent with the notion of hypofrontality in PwPP, which may contribute to psychiatric symptoms. We also found that higher BMI was correlated with higher glucose levels in OCC, but not PFC. Higher glucose levels might reflect metabolic differences (ie, lower glucose utilization in the brain) in PwPP per se, or could be a secondary effect of medication. Metabolic side effects including weight gain, higher blood glucose, and type 2 diabetes mellitus are common in individuals taking second generation antipsychotic medications.^90^^,^^91^ However, metabolic differences have also been observed in PwPP who have not taken antipsychotics.^92^^,^^93^

At the inception of our study in 2017, there were no reports of higher glucose levels among PwPP within the MRS literature, to our knowledge. However, similar results have recently been reported by Wijtenburg and colleagues.^8^ Using an ultra-short echo time (6.5 ms) STEAM sequence at 3 T, they found higher glucose levels in medial occipital cortex in people with schizophrenia (*n* = 16) versus controls (*n* = 19). Higher occipital glucose was correlated with poorer performance on the Brief Visuospatial Memory Test, and abnormal insulin resistance biomarker levels in their study. Thus, our observation that higher brain glucose was associated with impairment in a cluster of psychiatric, cognitive, and visual task performance measures appears consistent with these recent findings.^8^ As glucose was not among the 6 metabolites we examined a priori, we applied a statistically conservative FDR correction (for 10 comparisons); our observation of higher PFC glucose in PwPP vs. controls was marginally significant after this correction. Quantification of glucose via MRS is complicated by overlap with resonances from other metabolites, relatively low concentrations of glucose in the brain (~1 mM),^94^ and presence of glucose in the CSF.^83^^,^^84^ Both our study and that of Wijtenburg and colleagues^8^ used ultra-short TE (≤8 ms) STEAM sequences, which may facilitate quantification of glucose by minimizing signal loss due to *T_2_* decay and phase dispersion from *J* evolution. For this reason, it is possible that other previous MRS studies using sequences with longer TEs may not have observed higher levels of brain glucose in PwPP vs. controls. Additionally, our ability to detect group differences in glucose levels may have been facilitated by our fitting approach, and / or our use of a custom-built radiofrequency head coil, which yielded high data quality (eg, SNR ≥ 40; Supplementary Table 2).

Transverse relaxation time constants (*T_2_*) for some metabolites, including NAA, may be shorter in PwPP,^52^^,^^58–61^ which may reflect increased intracellular interactions between metabolites and macromolecules. At sufficiently long echo times (TEs), shorter *T_2_* would lead to smaller metabolic signals in PwPP, even in the absence of any true difference in concentration. In the current study, we used an ultra-short (8 ms) TE sequence in order to address this concern. Our observation that OCC NAA levels are lower in PwPP and relatives (and perhaps to a lesser extent in PFC for PwPP) indicates that NAA levels in the brain may indeed be lower in these groups, beyond confounding effects of shorter *T_2_*, suggesting a true impairment in neuronal health and metabolic function.^43^^,^^52^ Our findings also suggest that lower OCC NAA may be linked to genetic liability for psychosis, as expressed by first-degree biological relatives, who share on average 50% of their genes with a person with a psychotic disorder. However, the difference in OCC NAA between relatives vs. controls was not significant when age, sex, and BMI were included as covariates, and older age was associated with lower NAA levels. Thus, we cannot rule out the possibility that lower NAA levels in relatives may have been driven by the fact that the relatives tended to be older on average compared to controls.

NAA plays multiple roles in the human brain, which include providing storage for acetyl units that are used in myelin synthesis, regulating osmotic pressure, and supporting neural metabolic functions.^95^ NAA levels are thought to reflect the level of neuronal metabolic activity and / or neuronal density within a given brain region.^95^ As NAA synthesis is coupled to glucose metabolism,^96^ higher glucose and lower NAA levels in OCC may reflect a shared metabolic difference in PwPP. Lower occipital NAA has been observed previously in people with psychotic disorders including schizophrenia.^18^ Our study builds upon these findings by showing this deficit is present in a relatively large, trans-diagnostic group of PwPP, as well as biological relatives, assessed at 7 T. To date, comparatively few studies have examined NAA levels in biological relatives of PwPP; these have generally shown lower NAA concentrations for relatives versus controls across several brain regions.^21–23^^,^^27^

NAA is converted to NAAG through the addition of glutamate. NAAG is a peptide neurotransmitter that is thought to act as an agonist at metabotropic glutamate receptors (ie, mGluR2/3,^97^^,^^98^ but see^99^ for conflicting results). Modulation of glutamatergic NMDA receptors by NAAG has also been suggested, but this has been disputed.^100^ The ability to reliably quantify NAAG using MRS is hampered by its relatively low concentration in the human brain, and spectral overlap with other metabolites present at higher concentrations, specifically NAA.^94^ Often, these 2 metabolites are quantified together (NAA + NAAG). Here, separate quantification was facilitated by our use of 7 T MRS, and our high spectral quality (in terms of linewidth). Although the precise roles of NAAG as a neurotransmitter remain under investigation, differences in NAAG levels have been observed in schizophrenia.^86^^,^^101^ Our initial results suggested lower NAAG levels in PFC for PwPP as compared to relatives and controls, and a similar trend in OCC, which would be consistent with abnormal glutamatergic functioning.^2^^,^^102^ However, differences in NAAG were no longer significant after demographic factors (eg, age) were included as covariates, raising the possibility that NAAG differences may be explained by demographics.

With regard to specific diagnostic categories, we found lower OCC NAA among people with bipolar disorder vs. controls, and a trend in the same direction for people with schizophrenia. This suggests that lower NAA levels in this region may be a shared feature of both bipolar disorder with psychotic features and schizophrenia. *Myo*-inositol levels in OCC were lower among people with bipolar disorder vs. schizophrenia and controls. Previous MRS studies have reported reduced *myo*-inositol levels in bipolar disorder, which may be related to mood symptoms across the psychosis spectrum, and / or medication effects.^103^^,^^104^

We acknowledge a number of limitations of our study. Because there are concerns regarding the validity and reliability of traditional psychiatric diagnostic categories,^105^^,^^106^ we chose to adopt a transdiagnostic approach to studying neurochemistry in PwPP. This necessarily limited our ability to make comparisons between diagnostic groups, due to limitations on sample size. We had twice as many people assigned female versus male at birth in the relative group, and this group tended to be older in comparison to the other 2 groups. This limited our ability to exclude confounds of age and sex from our analyses of data from relatives. Relatives included individuals with and without current psychiatric diagnoses other than psychotic disorders (eg, depression, anxiety, substance use). Although this diagnostic heterogeneity reflects the diversity among first-degree biological relatives of PwPP, it also complicates interpretation of group comparisons involving relatives. For example, major depressive disorder may be associated with lower glutamate + glutamine levels in PFC.^107^ We corrected our metabolite measurements based on the content of GM, WM, and CSF, which were quantified from structural MRI data acquired in a separate, earlier scanning session at 3 T. Therefore, we were unable to account for any potential changes in tissue composition across time between 3 T and 7 T scanning. Many of the PwPP reported taking clinically determined doses of antipsychotic medication (Table 1), and our results should be interpreted in light of possible medication effects. However, we saw no significant relationships between metabolite levels and chlorpromazine dose equivalents.

## Supplementary Material

Schallmo_MRS_supplement_sbag137
