# Supplementary material for: Neurometabolic Differences in Psychosis Observed with 7 T Magnetic Resonance Spectroscopy
Source: Schizophr Bull. 2026 Oct 28;52(6):sbag137. doi: 10.1093/schbul/sbag137 (PMC13716499; doi:10.1093/schbul/sbag137)
Supplement: Supplementary file 1 [file schallmo_mrs_supplement_sbag137.docx]

# Supplemental Information

“Neurometabolic differences in psychosis observed with 7 T MRS”

Michael-Paul Schallmo, Caroline Demro, Kyle W. Killebrew, Aislin A. Sheldon, Cheryl A. Olman, Scott R. Sponheim, Małgorzata Marjańska

*Schizophrenia Bulletin*

## Supplemental methods

### Participants

Participants were 18-59 years old, spoke English as a primary language, and did not have: a diagnosed learning disability or IQ < 70, current or past central nervous system disease, history of head injury with skull fracture or loss of consciousness > 30 minutes, alcohol or drug abuse within the past month, or dependence within the last 6 months, electroconvulsive therapy within the last year, tardive dyskinesia, visual or hearing impairment. All participants were able to fit into the MRI scanner comfortably and had a corrected Snellen visual acuity of 20/40 (logMAR = 0.3) or better. Participants were recruited as part of the Psychosis Human Connectome Project [1,2] through advertisements, word of mouth, the University of Minnesota, and the Minneapolis VA Medical Center. We assessed each participant’s capacity to provide informed consent using the University of California Brief Assessment of Capacity to Consent [3]. Most participants in the PwPP group were in the chronic phase of illness (Table 1). Six PwPP had been diagnosed within 5 years prior to participation. Age of onset was < 45 years for all but one PwPP. Previous publications that have included 7 T MRS data from this cohort were limited to describing the methods and data quality [2], or supplemental analyses of bi-variate correlations between GABA in OCC and specific visual task measures [4,5].

### MR scanner

We used an in-scanner motion tracking system from Metria Innovation Inc., (Milwaukee, WI) with a Moiré Phase Tracking marker attached to the participant's nose to monitor head motion during scanning. Experimenters monitored head motion in real time during the scan, and when motion > 5 mm was observed, MRS scans were repeated, and participants were encouraged to remain still.

### MR spectroscopy

STEAM sequence parameters were as follows: repetition time (TR) = 5 s, echo time (TE) = 8 ms, mixing time (TM) = 32 ms, hsinc pulse = 1.28 ms, transmitter frequency = 3 ppm, number of complex data points = 2048, spectral bandwidth = 6000 Hz, chemical shift displacement error = 4% per ppm. We used 3D outer volume suppression interleaved with variable power and optimized relaxation delay (VAPOR) water suppression [6]. We adjusted the transmit voltage for each VOI in order to calibrate the B_1_ field for the 90° pulse and the water suppression flip angles.

We collected 128 shots in the PFC VOI for an initial group of 30 participants to assess data quality. After confirming that our PFC data were of comparable quality to those from our OCC VOI, the number of shots was reduced to 96 for subsequent participants. Our frontal MRS coil was not available during the first 6 months of data collection, which meant that we were not able to collect PFC data from all participants. OCC data collection always preceded PFC. We acquired a partial sagittal *T*_1_-weighted anatomical scan during our MRS experiment, which we used to guide voxel placement. We were limited to a partial sagittal acquisition due to the constraints of the surface coil used for MRS. *T*_1_ scan parameters collected during the MRS experiment were as follows: TR = 2500 ms, TE = 3.2 ms, echo spacing = 7.9 ms, flip angle = 5°, resolution = 1.3 mm isotropic, partial Fourier = 6/8, 64 mid-sagittal slices, field of view = 160 x 160 mm^2^.

### Visual tasks

We collected data from the visual tasks described below as part of the Psychosis Human Connectome Project. For full details, please see our recent publication [2]. Data were collected outside the scanner, prior to MR scanning, as part of the same study visit. Tasks were performed using PsychoPy [7] on a Mac Pro with a linearized FlexScan SX2462W monitor (60 Hz refresh rate, mean luminance = 61.2 cd/m^2^, viewing distance 70 cm) and a Bits# stimulus processor.

The first visual paradigm was designed to measure contrast discrimination thresholds for both isolated grating stimuli, as well as gratings presented in the context of a high contrast surrounding annular grating [5,8-12]. The former provided a measure of contrast sensitivity, whereas the latter measured the modulation of contrast discrimination by the surrounding grating (i.e., surround suppression). Eight stimulus conditions were used. The first seven included target gratings without surrounds at 0, 0.6, 1.25, 2.5, 5, 10, and 20% pedestal contrast. The eighth used 10% contrast targets with 100% contrast surrounds. Targets and surrounds (when present) were presented on both sides of a central fixation mark at 3° eccentricity (target diameter = 2°, surround inner and outer diameter = 2.5° and 4°, respectively, spatial frequency = 1.1 cycles/°, contrast reversing at 4 Hz). On each trial, targets appeared for 800 ms, followed by an unlimited response period. A contrast increment was added to the target grating on one side (randomized) on every trial. The participants’ task was to indicate which target was higher contrast (left or right). The increment value was controlled by a Psi adaptive staircase across trials [13]. This allowed us to find an increment value at which the participants could reliably discriminate which target was higher contrast. Each staircase included 30 trials, and there were 3 independent staircases per stimulus condition. Conditions were presented in separate blocks. Threshold values were calculated at 80% accuracy by fitting the staircase data for each condition with a Weibull function. For this study, we calculated the average contrast discrimination threshold by taking the geometric mean of the threshold values from the 7 conditions without surrounds. We calculated a contrast surround suppression index by taking the ratio of the thresholds for targets at 10% contrast with surrounds / targets without surrounds (at 10% contrast).

The second visual task focused on contour object perception [14]. Discrete visual elements (i.e., Gabors, *SD* = 0.067°, spatial frequency = 5 cycles/°) composed an egg-shaped contour (15 elements, egg was 5.9° wide x 4.7° high). Participants had to perceptually integrate the contour elements in order to discriminate the directionality of the egg shape (i.e., pointing left or right) within a field of 170 randomly oriented background distractor elements [15-18]. Data from two types of contour object perception trials were used in the current study. In the first type, the orientation of the contour elements was perfectly aligned with the axis of the egg-shaped contour. In the second type of trials, orientation jitter was added such that the Gabors that composed the contour were miss-aligned with the contour axis; this was done in order to make the directionality of the contour more difficult to perceive. Jitter was controlled across trials using a Psi adaptive staircase, in order to find the jitter level at which participants could reliably discriminate left- versus right-facing contours. Staircases for the jittered contour condition included 30 trials, and there were 3 independent staircases in each of two block (6 total staircases). Jitter threshold values at 70% accuracy were obtained by fitting the staircase data from each block with a Weibull function. There were also 20 trials of the aligned contour condition in each block (40 total). For this study, we calculated the average accuracy for contour object discrimination across trials in the aligned contour condition. We also calculated the average jitter threshold across the two blocks.

Our third visual task focused on a bi-stable visual structure-from-motion paradigm known as the rotating cylinder illusion [4,19,20]. In this task, participants viewed an array of 400 squares (0.25° wide, 200 black, 200 white) that moved horizontally across the screen in a manner that simulated the rotation of a 3-dimensional cylinder in depth (height = 10°, width = 7°, rotation speed = 90°/s). Because the stimulus lacked depth cues, the direction of rotation (i.e., clockwise or counterclockwise in depth) was ambiguous, which caused the image to appear to alternate between these two perceptual interpretations spontaneously in a bi-stable manner. Participants reported their initial percept and all subsequent perceptual switches by pressing a key to indicate which direction the front face of the cylinder appeared to rotate. Participants viewed and responded to a continuously rotating image for 2 minutes in each of 5 task blocks. Based on the timing of reported perceptual switches, we calculated the average structure-from-motion bi-stable switch rate across blocks, as well as the coefficient of variance (i.e., the standard deviation of the percept durations, divided by the mean).

All three of our visual tasks included catch trials in order to assess the participants’ ability to understand and carry out the task as instructed. Data from participants who failed to achieve sufficiently high performance in these catch trials were excluded; see our recent paper for full details [2].

Supplemental Figure 1. Timeline of data collection. Clinical and 3 T data were each collected as part of larger separate experimental sessions, as described in [1]. 3 T session also included diffusion weighted MRI, resting state functional MRI, and cognitive task fMRI. Visual task and MRS data were acquired as part of a larger 7 T session, as described in [2]. 7 T session also included visual task fMRI, which always followed MRS. SCID: Structured Clinical Interview for DSM-IV-TR, BACS: Brief Assessment of Cognition in Schizophrenia, SGI: Sensory Gating Inventory, SPQ: Schizotypal Personality Questionnaire, PID-5: Personality Inventory for the DSM-5, BPRS: Brief Psychiatric Rating Scale, CSS: Contrast Surround Suppression, COP: Contour Object Perception, SFM: structure-from-motion.

### Data processing

We processed our MRS data using the *matspec* toolbox ([github.com/romainVala/matspec](https://github.com/romainVala/matspec)) in MATLAB. In the rare cases where we found that frequency and / or phase correction failed (based on visual inspection), we manually excluded individual shots. We also manually excluded a small number of full data sets (OCC = 3, PFC = 5) with significant artifacts (e.g., failed water suppression, defined as a water peak > NAA peak). We used a basis set from a previous study [21], which included 18 metabolites: ascorbic acid, aspartic acid, creatine, γ-aminobutyric acid (GABA), glucose, glutamate, glutamine, glutathione, glycerophosphorylcholine, lactate, *myo*-inositol, *N*-acetyl-aspartate (NAA), *N*-acetyl-aspartylglutamate (NAAG), phosphocreatine, phosphorylcholine, phosphorylethanolamine, *scyllo*-inositol, taurine, in addition to lipids and macromolecules. The glucose basis spectrum was generated with 36% alpha-glucose and 64% beta-glucose. Macromolecule signals included in the basis set were measured using inversion-recovery experiments [22] performed in the OCC of 4 healthy adults [21].

Tissue fractions (gray matter, white matter, and CSF) were obtained from *T*_1_- and *T*_2_-weighted anatomical scans acquired at 3 T in a separate scanning session [1], and then processed through the Human Connectome Project minimal preprocessing pipeline version 3.22.0 [23]. This was necessary because we used a surface coil designed for MRS at 7 T, which did not provide full coverage for anatomical imaging of the whole brain. To obtain tissue fraction values for each MRS VOI, we aligned the partial sagittal *T*_1_-weighted anatomical data from the 7 T MRS session to the whole brain *T*_1_ scan acquired at 3 T. Water scaling correction values are reported in Supplemental Table 1. We did not correct for *T*_1_ and *T*_2_ relaxation times for metabolites, as these effects are expected to be very small given the long TR (5 s) and ultra-short TE (8 ms).

Supplemental Table 1. Water scaling correction parameters. Columns show the water content (%), *T*_1_ and *T*_2_ relaxation time constants (ms) for gray matter (GM), white matter (WM), and cerebrospinal fluid (CSF; rows). These values are based on previous work [21].

|  | **Water content** | ***T*_1_ relaxation time constant (ms)** | ***T*_2_ relaxation time constant (ms)** |
| --- | --- | --- | --- |
| GM | 80% | 2130 | 50 |
| WM | 71% | 1220 | 55 |
| CSF | 97% | 4425 | 141 |

We used the following MRS data quality metrics. First, we set a maximum value for water linewidth of 15 Hz, to ensure sufficient shim quality and *B*_0_ homogeneity within our VOIs. This value was determined *a priori*, and was assessed during each scan using tools from the *Spectroscopy* tab in the Siemens console software. In general, if we obtained a water linewidth > 15 Hz, the VOI was repositioned and re-shimmed. However, in a small number of cases (n = 3), MRS data were acquired with a water linewidth > 15 Hz (and subsequently excluded). We also used two *post-hoc* data quality metrics provided by LCModel. Based on visual inspection of our LCModel data, we set maximum values for spectrum linewidth = 5 Hz and SNR = 40. Data sets that failed to meet one or more of these quality metrics were excluded.

LCModel quantifies SNR based on the maximum signal amplitude (i.e., of the NAA peak) divided by two times the root mean squared value of the residuals. Note that this means lower SNR values would be expected in a clinical population for which NAA levels are lower (e.g., PwPP), even without any difference in noise. After exclusion, we saw no significant differences in data quality across groups (Supplemental Table 2). Data quality metrics were also similar across OCC and PFC VOIs. Thus, we do not expect the larger size of the VOI in PFC to have a dramatic effect on quantification of metabolites, as compared to OCC.

Supplemental Table 2. Summary of MRS data quality. Data are presented for each VOI in each group as mean (*SD*). Note that for LCModel signal-to-noise ratio (LCM SNR) in OCC, lower SNR may be explained by lower NAA levels in PwPP, absent any difference in noise.

| **Data quality metric** | **Controls** | **Relatives** | **PwPP** | **Statistics** |
| --- | --- | --- | --- | --- |
| OCC H_2_O linewidth | 12.9 (0.9) | 13.2 (0.9) | 13.2 (0.8) | *X*^2^_2, n = 139_ = 2.24, *p* = 0.3 |
| OCC LCM linewidth | 4.03 (0.47) | 4.13 (0.45) | 4.21 (0.39) | *X*^2^_2, n = 141_ = 3.35, *p* = 0.19 |
| OCC LCM SNR | 54.2 (4.7) | 53.2 (5.4) | 51.9 (4.6) | *X*^2^_2, n = 141_ = 5.77, *p* = 0.056 |
| PFC H_2_O linewidth | 12.9 (1.1) | 12.6 (1.2) | 12.6 (1.2) | *X*^2^_2, n = 109_ = 1.54, *p* = 0.5 |
| PFC LCM linewidth | 4.01 (0.41) | 4.12 (0.50) | 4.08 (0.44) | *X*^2^_2, n = 112_ = 1.10, *p* = 0.6 |
| PFC LCM SNR | 51.9 (4.1) | 51.2 (5.0) | 51.1 (6.3) | *X*^2^_2, n = 112_ = 0.56, *p* = 0.8 |

### Data analysis and statistics

Our supplemental analyses examined whether demographic or other factors might account for the observed results in metabolite levels reported in the main text. For each of the 6 metabolites we examined *a priori*, we used linear mixed effects models to assess group differences in metabolite levels in OCC and PFC, with age, sex, and body mass index (BMI) included as covariates. Because BMI differed significantly across groups (Table 1), we chose to include BMI as a covariate, due to concerns that obesity may be associated with differences in brain metabolite levels (especially glucose). For glucose in OCC and PFC, variance was not equal between groups (Figure 3C & D). Therefore, instead of linear mixed effects models, we used Kruskal-Wallis 1-way non-parametric ANOVAs and Spearman correlations.

We asked whether differences in metabolites might be related to differences in tissue composition (e.g., lower GM in PwPP) [24,25]. For this purpose, we calculated the proportion of gray matter (GM), normalized by the summed proportions of gray matter and white matter (WM), as follows:

$$Normalized GM=\frac{\% GM}{(\% GM+\% WM)}$$

Supplemental Equation 1

The proportions of GM, WM, and CSF within each VOI were obtained from FreeSurfer [26]. We assessed group differences in normalized GM and CSF fractions using Kruskal-Wallis 1-way non-parametric ANOVAs. We also examined Spearman correlations between these tissue composition metrics and metabolite levels. Note that this was done in addition to correcting metabolite levels for tissue content within the VOIs.

To examine whether antipsychotic medication levels among PwPP may have been associated with metabolite concentrations, we quantified chlorpromazine (CPZ) dose equivalents using established methods [27,28], and used Spearman correlations. We did not collect data on the duration of antipsychotic use.

For our hierarchical clustering analysis [29], we calculated the absolute correlation distance for each pair of variables, as one minus the absolute value of the Spearman correlation. We used Spearman rank correlations to avoid assumptions of normally distributed variables with linear relationships. Note that for the clustering analyses, we included data for visual tasks and clinical measures from all individuals who participated in our 7 T experimental protocols (and were not excluded for eligibility issues; total n = 153, 43 controls, 44 relatives, 66 PwPP, i.e., an additional 4 relatives and 8 PwPP who did not provide useable MRS data). For experimental details and demographic information from the full study, see our recent publication [2]. Cluster analyses were performed with the *linkage.m* and *cluster.m* functions in MATLAB. Linkage was computed using the unweighted average distance.

As a goal of the current study was to examine associations between impaired visual functioning and brain chemistry in PwPP, our first hierarchical clustering analysis focused on relationships with metabolite levels in OCC. Because understanding neurochemical markers in prefrontal regions that may be associated with higher-level cognitive or perceptual impairments was a secondary goal, and to mitigate the risk of false positive results due to multiple comparisons, we chose to perform a second hierarchical analysis examining metabolite levels from both OCC and PFC (but not data from PFC alone).

We used principal component analyses to determine the appropriate number of clusters for each of our hierarchical clustering analyses. Scree plots (i.e., percent variance explained per component) are shown in Supplemental Figure 12. The appropriate number of clusters (*k*) was chosen according to the elbow method. Based on the PCA results, we chose to examine *k* = 6 clusters for the OCC clustering analysis presented in Figure 4. However, because of the hierarchical nature of the approach, results were similar when using a smaller or larger number of clusters (i.e., *k* = 3 to 7; Supplemental Figure 7). Multiple ‘elbows’ were observed for OCC clustering for data from all participants, and for OCC+PFC clustering for data from all participants. We chose to include the higher number of clusters in both cases (i.e., *k* = 6 and 7, respectively; Supplemental Figure 12A & C). A single, more gradual ‘elbow’ was observed for OCC clustering for data from PwPP only; we chose *k* = 4 as the midpoint (Supplemental Figure 12B).

To assess the statistical significance for our clustering results, we performed permutation analyses: 1) with data randomly shuffled across participants prior to clustering (to generate a null distribution of *r* values), and 2) with variables randomly shuffled across clusters (to generate a null distribution of clusters based on observed *r* values). For these analyses, we calculated the mean of all *r* values within each cluster after taking the absolute value. This was repeated across 1000 iterations. We quantified significance (i.e., *p* value) based on the proportion of permuted *r* values generated under the null distribution that were larger than the observed mean absolute cluster *r* values. To quantitatively examine the similarity between our OCC clustering results from all participants vs. those from PwPP only, we performed an additional permutation analysis. Here, we determined the likelihood of observing a similar cluster (i.e., ≥ 7 variables, of which ≥ 87.5% were part of the red cluster that included glucose in Figure 4), with data (from PwPP only) shuffled across participants.

Supplemental Table 3. List of variables for OCC hierarchical clustering analysis. Visual tasks are from [2] and are abbreviated as follows: contrast surround suppression (CSS), contour object perception (COP), structure-from-motion (SFM).

| **Variable #** | **Variable type** | **Variable name** |
| --- | --- | --- |
| 1 | Visual task | Contrast discrimination (CSS) threshold |
| 2 | Visual task | Contrast surround suppression (CSS) index |
| 3 | Visual task | Contour object discrimination (COP) accuracy |
| 4 | Visual task | Contour object (COP) jitter threshold |
| 5 | Visual task | Structure-from-motion (SFM) bi-stable switch rate |
| 6 | Visual task | Structure-from-motion (SFM) bi-stable coefficient of variance |
| 7 | Clinical measure | BPRS total score |
| 8 | Clinical measure | BACS *Z*-score |
| 9 | Clinical measure | SGI total score |
| 10 | Clinical measure | SPQ total score |
| 11 | Clinical measure | PID-5 psychoticism factor |
| 12 | Clinical measure | BPRS hallucination score |
| 13 | Clinical measure | Snellen visual acuity |
| 14 | Clinical measure | Mars letter contrast sensitivity |
| 15 | MRS measure | OCC ascorbic acid |
| 16 | MRS measure | OCC aspartic acid |
| 17 | MRS measure | OCC total creatine |
| 18 | MRS measure | OCC GABA |
| 19 | MRS measure | OCC glucose |
| 20 | MRS measure | OCC glutamine |
| 21 | MRS measure | OCC glutamate |
| 22 | MRS measure | OCC total choline |
| 23 | MRS measure | OCC glutathione |
| 24 | MRS measure | OCC *myo*-inositol |
| 25 | MRS measure | OCC macromolecules |
| 26 | MRS measure | OCC NAA |
| 27 | MRS measure | OCC NAAG |
| 28 | MRS measure | OCC phosphorylethanolamine |
| 29 | MRS measure | OCC *scyllo*-inositol |
| 30 | MRS measure | OCC taurine |

Supplemental Table 4. List of variables for OCC+PFC hierarchical clustering analysis. Visual tasks are from [2] and are abbreviated as follows: contrast surround suppression (CSS), contour object perception (COP), structure-from-motion (SFM).

| **Variable #** | **Variable type** | **Variable name** |
| --- | --- | --- |
| 1 | Visual task | Contrast discrimination (CSS) threshold |
| 2 | Visual task | Contrast surround suppression (CSS) index |
| 3 | Visual task | Contour object discrimination (COP) accuracy |
| 4 | Visual task | Contour object (COP) jitter threshold |
| 5 | Visual task | Structure-from-motion (SFM) bi-stable switch rate |
| 6 | Visual task | Structure-from-motion (SFM) bi-stable coefficient of variance |
| 7 | Clinical measure | BPRS total score |
| 8 | Clinical measure | BACS *Z*-score |
| 9 | Clinical measure | SGI total score |
| 10 | Clinical measure | SPQ total score |
| 11 | Clinical measure | PID-5 psychoticism factor |
| 12 | Clinical measure | BPRS hallucination score |
| 13 | Clinical measure | Snellen visual acuity |
| 14 | Clinical measure | Mars letter contrast sensitivity |
| 15 | MRS measure | OCC ascorbic acid |
| 16 | MRS measure | OCC aspartic acid |
| 17 | MRS measure | OCC total creatine |
| 18 | MRS measure | OCC GABA |
| 19 | MRS measure | OCC glucose |
| 20 | MRS measure | OCC glutamine |
| 21 | MRS measure | OCC glutamate |
| 22 | MRS measure | OCC total choline |
| 23 | MRS measure | OCC glutathione |
| 24 | MRS measure | OCC *myo*-inositol |
| 25 | MRS measure | OCC macromolecules |
| 26 | MRS measure | OCC NAA |
| 27 | MRS measure | OCC NAAG |
| 28 | MRS measure | OCC phosphorylethanolamine |
| 29 | MRS measure | OCC *scyllo*-inositol |
| 30 | MRS measure | OCC taurine |
| 31 | MRS measure | PFC ascorbic acid |
| 32 | MRS measure | PFC aspartic acid |
| 33 | MRS measure | PFC total creatine |
| 34 | MRS measure | PFC GABA |
| 35 | MRS measure | PFC glucose |
| 36 | MRS measure | PFC glutamine |
| 37 | MRS measure | PFC glutamate |
| 38 | MRS measure | PFC total choline |
| 39 | MRS measure | PFC glutathione |
| 40 | MRS measure | PFC *myo*-inositol |
| 41 | MRS measure | PFC macromolecules |
| 42 | MRS measure | PFC NAA |
| 43 | MRS measure | PFC NAAG |
| 44 | MRS measure | PFC phosphorylethanolamine |
| 45 | MRS measure | PFC *scyllo*-inositol |
| 46 | MRS measure | PFC taurine |

## Supplemental results

### Covariates

Because our groups differed on factors such as age and BMI (see Table 1), we performed follow-up analyses to examine whether these may have influenced our observations. We found that NAA levels in OCC were significantly associated with age (linear mixed-effects model, parameter estimate [SE] = ‑0.014 [0.004], *t*_156_ = -3.38, *p* = 9 x 10^-4^), but not sex assigned at birth (parameter estimate [SE] = -0.11 [0.09], *t*_156_ = -1.20, *p* = 0.2) or BMI (parameter estimate [SE] = -0.003 [0.009], *t*_156_ = -0.32, *p* = 0.7). With these covariates included, NAA was significantly lower in PwPP compared with controls (parameter estimate [SE] = ‑0.25 [0.10], *t*_124_ = -2.37, *p* = 0.019; Supplemental Table 7). However, the difference between controls and relatives was not significant (parameter estimate [SE] = -0.18 [0.14], *t*_75_ = -1.25, *p* = 0.2). Thus, we cannot rule out the possibility that lower NAA levels in OCC in relatives in particular may have been driven by the fact that this group was older than the controls on average.

Non-parametric tests suggested NAAG in OCC was lower among PwPP vs. relatives (*Χ*^2^_1, n = 98_ = 4.01, *p* = 0.045), and trended lower in PwPP vs. controls (*Χ*^2^_1, n = 79_ = 3.31, *p* = 0.069). However, with age, sex, and BMI included as covariates, we saw no significant differences between groups for NAAG levels in OCC (linear mixed-effects models, controls vs. PwPP: parameter estimate [SE] = -0.044 [0.044], *t*_124_ = -1.00, *p* = 0.3; relatives vs. PwPP: parameter estimate [SE] = -0.048 [0.051], *t*_110_ = -0.95, *p* = 0.3). NAAG levels in OCC were significantly associated with age (parameter estimate [SE] = 0.006 [0.001], *t*_156_ = 3.51, *p* = 6 x 10^‑4^), but not with sex assigned at birth (parameter estimate [SE] = -0.057 [0.037], *t*_156_ = 1.54, *p* = 0.13) or BMI (parameter estimate [SE] = -2 x 10^-4^ [0.003], *t*_156_ = -0.05, *p* = 1.0).

We performed the same analyses for NAAG levels in PFC, which were also significantly associated with age (linear mixed-effects model, parameter estimate [SE] = 0.006 [0.001], *t*_113_ = 4.43, *p* = 2 x 10^-5^), but not sex assigned at birth (parameter estimate [SE] = 0.038 [0.033], *t*_113_ = 1.13, *p* = 0.2) or BMI (parameter estimate [SE] = 0.002 [0.003], *t*_113_ = 0.53, *p* = 0.6). However, after accounting for these variables, the difference in NAAG between PwPP and controls was not significant (parameter estimate [SE] = -0.06 [0.04], *t*_87_ = -1.41, *p* = 0.16; Supplemental Table 7). Thus, we cannot exclude differences in these factors as a potential explanation for our observation of lower NAAG in PFC in PwPP.

To assess the possible contribution of age, sex assigned at birth, and BMI to other metabolite levels examined *a priori*, we conducted follow-up analyses using linear mixed-effects models with these factors included as co-variates. We observed a trend toward lower glutamate levels in OCC among PwPP versus controls, which did not reach statistical significance (linear mixed-effects model, parameter estimate [SE] = -0.183 [0.094], *t*_124_ = -1.95, *p* = 0.054; Supplemental Table 7). We saw a similar trend for lower OCC glutamate in PwPP vs. relatives (parameter estimate [SE] = -0.169 [0.095], *t*_110_ = -1.77, *p* = 0.079). In PFC, we saw trends toward lower GABA (parameter estimate [SE] = -0.096 [0.055], *t*_87_ = -1.75, *p* = 0.083), glutathione (parameter estimate [SE] = -0.05 [0.03], *t*_87_ = -1.67, *p* = 0.098), & NAA (parameter estimate [SE] = -0.326 [0.166], *t*_87_ = -1.97, *p* = 0.052) in PwPP versus controls, which did not reach significance. Other group differences were not significant with covariates included (see Supplemental Table 7).

We saw no significant differences in glucose levels between people assigned male or female at birth in either OCC or PFC (*Χ*^2^_1_ values < 0.09, *p* values > 0.7; Supplemental Table 9). There were also no significant correlations between glucose levels in these regions and age (Spearman’s *r* values < 0.10, *p* values ≥ 0.3; Supplemental Table 9). We did observe a significant correlation between higher glucose levels in OCC and higher BMI (*r*_120_ = 0.26, *p* = 0.004; Supplemental Figure 4A), but this relationship was not significant in PFC (*r*_92_ = 0.13, *p* = 0.2; Supplemental Figure 4B). Correlations between glucose levels in OCC and BMI were comparable in magnitude (but not significant) within our group of relatives when considered alone (*r*_31_ = 0.29, *p* = 0.10), but were numerically lower in PwPP (*r*_50_ = 0.13, *p* = 0.4). This is notable as BMI, but not glucose levels in OCC, were significantly higher among relatives vs. controls (see main text; Table 1). Although we cannot rule out an influence of higher BMI on glucose levels in PwPP and / or relatives, BMI alone may not fully account for the observed group differences in brain glucose levels.

### Duration of psychotic illness

In both OCC and PFC, we saw significant positive correlations between NAAG levels and years of illness among PwPP (Spearman correlation, OCC: *r*_45_ = 0.32, *p* = 0.027; PFC: *r*_40_ = 0.35, *p* = 0.024). However, we observed a similar relationship between age and NAAG levels across all participants in both OCC and PFC (linear mixed-effects models, OCC: parameter estimate [SE] = 0.006 [0.002], *t*_113_ = 3.51, *p* = 6 x 10^-4^; PFC: parameter estimate [SE] = 0.006 [0.001], *t*_113_ = 4.43, *p* = 2 x 10^-5^). Because duration of illness and age were highly correlated in our sample of PwPP (*r*_40_ = 0.74, *p* = 2 x 10^-8^), it is unclear to what extent the duration of psychotic illness may impact NAAG levels, independent of effects of age. Other metabolites were not significantly correlated with illness duration (see Supplemental Table 8 & 9).

### Medication effects

In PwPP, we found no significant associations between chlorpromazine equivalent doses [27,28] and NAA in OCC (*r*_56_ = 0.09, *p* = 0.5) or NAAG in PFC (*r*_48_ = 0.02, *p* = 0.9; Supplemental Table 8). Nor were there any significant correlations between antipsychotic medication scores and glucose levels in PwPP (|*r*| values < 0.14, *p* values ≥ 0.3; Supplemental Table 9). Thus, we did not find evidence to suggest a direct link between antipsychotic medication levels and the observed group differences in brain metabolites. CPZ levels were also not significantly correlated with any of the other metabolites we examined *a priori* in OCC or PFC (see Supplemental Table 8 & 9).

Participants took their prescribed medications according to their typical schedule, and were not asked to withhold or alter their medication regimen. We did not observe any significant effects of benzodiazepine use on GABA levels in OCC (linear mixed effect model, parameter estimate [SE] = -0.02 [0.05], *t*_155_ = -0.40, *p* = 0.7) or in PFC (parameter estimate [SE] = -0.09 [0.06], *t*_112_ = -1.64, *p* = 0.104).

### Tissue composition

In OCC, we found a significant correlation between higher NAA and a higher normalized proportion of GM (*r*_137_ = 0.38, *p* = 4 x 10^-6^). However, groups did not differ significantly in normalized GM (*X*^2^_2, n = 141_ = 4.70, *p* = 0.095), suggesting differences in brain structure between groups may not fully account for lower OCC NAA in relatives and PwPP. NAA levels in OCC were not significantly correlated with CSF proportion (*r*_137_ = 0.16, *p* = 0.057), and CSF proportion did not differ across groups (*X*^2^_2, n = 141_ = 0.08, *p* = 1.0).

In PFC, higher NAAG levels correlated with a lower normalized GM (*r*_108_ = -0.26, *p* = 0.005), but not with CSF (*r*_108_ = 0.14, *p* = 0.14). There were no significant group differences in GM or CSF in PFC (*X*^2^_2_ values < 4.08, *p* values > 0.13), suggesting differences in brain structure may not account for higher PFC NAAG in PwPP.

Because glucose is present in CSF [30-32], we investigated whether group differences in CSF fraction may have influenced our results. Higher glucose levels correlated with higher proportions of CSF in PFC (*r*_110_ = 0.29, *p* = 0.002) as expected [30-32], but not in OCC (*r*_139_ = 0.13, *p* = 0.12). However, the lack of significant group differences in CSF in either VOI (*Χ*^2^_2_ values < 3.2, *p* values > 0.2) suggests that the proportion of CSF may not explain the observed group differences in glucose (Figure 3C & D). Correlations between glucose and normalized GM were not significant (*r* values < 0.11, *p* values > 0.2).

### Hierarchical clustering

To assess the extent to which our OCC clustering results from all participants were similar to those from PwPP alone, we repeated our hierarchical clustering analysis (main text; Figure 4) using data from PwPP only. We saw similar results for data from PwPP only; OCC glucose (red font) again clustered with visual task, cognition, and symptom measures (purple cluster in Supplemental Figure 8 & 9). Specifically, 7 of the 8 variables within this cluster also clustered together in our analysis of OCC data from all participants (i.e., BPRS, BACS, SGI, SPQ, PID-5 psychoticism, hallucinations, and glucose in OCC). A permutation test indicated that the similarity between these clustering results was statistically significant (with data shuffled across participants, *p* = 0.001). For data from PwPP only, the average correlation (*r* value) within the cluster that included glucose was also significantly higher than for shuffled data (permutation test with data shuffled across participants: *p* = 0.002; with variables shuffled across clusters: *p* = 0.002).

We also observed similar results for OCC+PFC clustering across all participants. Glucose in both OCC and PFC (red font) clustered with cognitive, symptom, and visual task measures (yellow cluster in Supplemental Figure 10 & 11). We note that NAAG levels from both OCC and PFC were also included in this cluster. Permutation analyses again showed that the average correlation (*r* value) within this cluster was significantly higher than for shuffled data (permutation test with data shuffled across participants: *p* < 0.001; with variables shuffled across clusters: *p* = 0.039).

## Supplemental figures and tables

Supplemental Figure 2. Additional metabolites in OCC. Group differences in these metabolites were not significant. Metabolites examined *a priori*: panels E-H, K; exploratory: panels A-D, I, J, L-N. C = controls, R = relatives, P = people with psychotic psychopathology. Thick lines show group medians, boxes show 25-75%, whiskers show 1.5x interquartile range, dots show individual data points. Metabolite abbreviations not defined in the main text are as follows: glutamate (Glu), glutamine (Gln), glutathione (GSH), ascorbic acid (Asc), aspartic acid (Asp), total choline (tCho), total creatine (tCr), glucose (Glc), macromolecules (MacM), *myo*-inositol (mIns), phosphorylethanolamine (PE), *scyllo*-inositol (sIns), taurine (Tau). For our exploratory analyses, we applied false discovery rate (FDR) correction for multiple comparisons.

Supplemental Figure 3. Additional metabolites in PFC. Group differences in these metabolites were not significant. Metabolites examined *a priori*: panels E-H, K; exploratory: panels A-D, I, J, L-N. C = controls, R = relatives, P = people with psychotic psychopathology. Thick lines show group medians, boxes show 25-75%, whiskers show 1.5x interquartile range, dots show individual data points. Metabolite abbreviations not defined in the main text are as follows: glutamate (Glu), glutamine (Gln), glutathione (GSH), ascorbic acid (Asc), aspartic acid (Asp), total choline (tCho), total creatine (tCr), glucose (Glc), macromolecules (MacM), *myo*-inositol (mIns), phosphorylethanolamine (PE), *scyllo*-inositol (sIns), taurine (Tau). For our exploratory analyses, we applied false discovery rate (FDR) correction for multiple comparisons.

Supplemental Table 5. Metabolite concentrations. Data are reported as means and standard deviations (SD) for occipital (OCC) and prefrontal (PFC) VOIs in controls (n = 43 in OCC, n = 29 in PFC), relatives (n = 40 in OCC, n = 33 in PFC), and PwPP (n = 58 in OCC, n = 50 in PFC). Units are millimolar (mM), except for macromolecules (arbitrary units).

| **Metabolite** | **Controls, mean** | **Controls, SD** | **Relatives, mean** | **Relatives, SD** | **PwPP, mean** | **PwPP, SD** |
| --- | --- | --- | --- | --- | --- | --- |
| OCC ascorbic acid | 1.67 | 0.18 | 1.59 | 0.16 | 1.62 | 0.20 |
| OCC aspartic acid | 1.45 | 0.38 | 1.32 | 0.45 | 1.23 | 0.41 |
| OCC total choline | 1.03 | 0.10 | 1.03 | 0.11 | 1.03 | 0.13 |
| OCC total creatine | 8.40 | 0.41 | 8.40 | 0.50 | 8.39 | 0.51 |
| OCC GABA | 1.08 | 0.17 | 1.07 | 0.20 | 1.06 | 0.21 |
| OCC glucose | 1.01 | 0.37 | 1.04 | 0.55 | 1.27 | 0.52 |
| OCC glutamate | 8.57 | 0.50 | 8.54 | 0.47 | 8.46 | 0.47 |
| OCC glutamine | 2.84 | 0.34 | 2.74 | 0.36 | 2.86 | 0.50 |
| OCC glutathione | 0.84 | 0.10 | 0.81 | 0.09 | 0.84 | 0.11 |
| OCC macromolecules | 0.078 | 0.003 | 0.078 | 0.003 | 0.077 | 0.003 |
| OCC *myo*-inositol | 7.16 | 0.59 | 7.01 | 0.69 | 6.94 | 1.01 |
| OCC NAA | 11.03 | 0.55 | 10.81 | 0.65 | 10.80 | 0.50 |
| OCC NAAG | 1.79 | 0.18 | 1.83 | 0.22 | 1.73 | 0.25 |
| OCC phosphoryl-  ethanolamine | 1.09 | 0.21 | 1.05 | 0.23 | 1.11 | 0.20 |
| OCC *scyllo*-inositol | 0.30 | 0.16 | 0.29 | 0.16 | 0.25 | 0.12 |
| OCC taurine | 1.64 | 0.25 | 1.62 | 0.22 | 1.64 | 0.29 |
| PFC ascorbic acid | 1.62 | 0.15 | 1.54 | 0.29 | 1.55 | 0.19 |
| PFC aspartic acid | 1.73 | 0.46 | 1.67 | 0.49 | 1.65 | 0.42 |
| PFC total choline | 1.74 | 0.17 | 1.73 | 0.21 | 1.67 | 0.23 |
| PFC total creatine | 8.72 | 0.56 | 8.68 | 0.83 | 8.58 | 0.62 |
| PFC glucose | 1.10 | 0.21 | 1.13 | 0.26 | 1.10 | 0.25 |
| PFC GABA | 0.81 | 0.41 | 0.93 | 0.45 | 1.15 | 0.56 |
| PFC glutamate | 9.98 | 0.51 | 10.07 | 0.59 | 9.94 | 0.67 |
| PFC glutamine | 3.57 | 0.45 | 3.43 | 0.39 | 3.57 | 0.63 |
| PFC glutathione | 1.21 | 0.12 | 1.17 | 0.13 | 1.20 | 0.13 |
| PFC macromolecules | 0.070 | 0.006 | 0.071 | 0.006 | 0.070 | 0.006 |
| PFC *myo*-inositol | 7.64 | 0.75 | 7.50 | 0.90 | 7.30 | 0.96 |
| PFC NAA | 10.05 | 0.56 | 10.03 | 0.57 | 9.97 | 0.60 |
| PFC NAAG | 1.26 | 0.18 | 1.29 | 0.19 | 1.18 | 0.17 |
| PFC phosphoryl-  ethanolamine | 1.94 | 0.25 | 1.95 | 0.27 | 2.05 | 0.26 |
| PFC *scyllo*-inositol | 0.25 | 0.12 | 0.25 | 0.17 | 0.20 | 0.11 |
| PFC taurine | 2.00 | 0.25 | 1.95 | 0.27 | 1.95 | 0.32 |

Supplemental Table 6. Cramér-Rao lower bound (CRLB) values. Data (in absolute units [mM], not percentages) are reported as means, standard deviations (SD), minimum (min.) and maximum (max.) for occipital (OCC) and prefrontal (PFC) VOIs across controls (C), relatives (R), and PwPP (P).

| **Metabolite** | **C, mean** | **C, SD** | **C, min** | **C, max** | **R, mean** | **R, SD** | **R, min** | **R, max** | **P, mean** | **P, SD** | **P, min** | **P, max** |
| --- | --- | --- | --- | --- | --- | --- | --- | --- | --- | --- | --- | --- |
| CRLB OCC  ascorbic acid (mM) | 0.17 | 0.01 | 0.15 | 0.21 | 0.17 | 0.01 | 0.14 | 0.20 | 0.17 | 0.01 | 0.14 | 0.20 |
| CRLB OCC  aspartic acid (mM) | 0.23 | 0.02 | 0.20 | 0.30 | 0.23 | 0.02 | 0.19 | 0.28 | 0.23 | 0.04 | 0.00 | 0.29 |
| CRLB OCC total  choline (mM) | 0.14 | 0.01 | 0.12 | 0.17 | 0.14 | 0.02 | 0.04 | 0.17 | 0.13 | 0.02 | 0.04 | 0.16 |
| CRLB OCC total  creatine (mM) | 0.43 | 0.04 | 0.36 | 0.56 | 0.44 | 0.05 | 0.35 | 0.54 | 0.43 | 0.04 | 0.34 | 0.53 |
| CRLB OCC  GABA (mM) | 0.14 | 0.01 | 0.12 | 0.17 | 0.14 | 0.01 | 0.12 | 0.17 | 0.14 | 0.01 | 0.11 | 0.17 |
| CRLB OCC  glucose (mM) | 0.22 | 0.01 | 0.19 | 0.26 | 0.22 | 0.01 | 0.19 | 0.26 | 0.22 | 0.02 | 0.18 | 0.26 |
| CRLB OCC  glutamate (mM) | 0.17 | 0.01 | 0.15 | 0.19 | 0.17 | 0.01 | 0.16 | 0.20 | 0.17 | 0.01 | 0.14 | 0.19 |
| CRLB OCC  glutamine (mM) | 0.14 | 0.01 | 0.11 | 0.18 | 0.14 | 0.01 | 0.11 | 0.17 | 0.14 | 0.01 | 0.11 | 0.17 |
| CRLB OCC  glutathione (mM) | 0.08 | 0.01 | 0.07 | 0.10 | 0.08 | 0.01 | 0.06 | 0.09 | 0.08 | 0.01 | 0.07 | 0.09 |
| CRLB OCC macro-  molecules (arb.) | 10x  10^-4^ | 4x  10^-4^ | 7x  10^-4^ | 23x  10^-4^ | 10x  10^-4^ | 4x  10^-4^ | 7x  10^-4^ | 17x  10^-4^ | 11x  10^-4^ | 4x  10^-4^ | 7x  10^-4^ | 21x  10^-4^ |
| CRLB OCC  *myo*-inositol (mM) | 0.14 | 0.01 | 0.12 | 0.17 | 0.14 | 0.01 | 0.12 | 0.18 | 0.14 | 0.02 | 0.11 | 0.19 |
| CRLB OCC  NAA (mM) | 0.18 | 0.05 | 0.10 | 0.24 | 0.18 | 0.05 | 0.10 | 0.24 | 0.18 | 0.05 | 0.10 | 0.24 |
| CRLB OCC  NAAG (mM) | 0.10 | 0.01 | 0.09 | 0.13 | 0.11 | 0.01 | 0.08 | 0.14 | 0.11 | 0.01 | 0.08 | 0.13 |
| CRLB OCC  phosphoryl-ethanolamine (mM) | 0.18 | 0.01 | 0.16 | 0.23 | 0.18 | 0.02 | 0.15 | 0.22 | 0.18 | 0.02 | 0.15 | 0.22 |
| CRLB OCC  *scyllo*-inositol (mM) | 0.04 | 3x  10^-3^ | 0.03 | 0.05 | 0.04 | 3x  10^-3^ | 0.03 | 0.04 | 0.04 | 5x  10^-3^ | 0.00 | 0.04 |
| CRLB OCC  taurine (mM) | 0.14 | 0.01 | 0.12 | 0.18 | 0.14 | 0.01 | 0.12 | 0.17 | 0.14 | 0.01 | 0.12 | 0.18 |
| CRLB PFC  ascorbic acid (mM) | 0.17 | 0.01 | 0.15 | 0.20 | 0.16 | 0.02 | 0.12 | 0.22 | 0.17 | 0.02 | 0.14 | 0.20 |
| CRLB PFC  aspartic acid (mM) | 0.22 | 0.02 | 0.19 | 0.27 | 0.22 | 0.04 | 0.00 | 0.30 | 0.23 | 0.02 | 0.19 | 0.28 |
| CRLB PFC total  choline (mM) | 0.13 | 0.03 | 0.03 | 0.15 | 0.13 | 0.02 | 0.04 | 0.16 | 0.12 | 0.03 | 0.04 | 0.16 |
| CRLB PFC total  creatine (mM) | 0.44 | 0.04 | 0.38 | 0.53 | 0.44 | 0.06 | 0.32 | 0.62 | 0.44 | 0.06 | 0.33 | 0.70 |
| CRLB PFC  GABA (mM) | 0.13 | 0.01 | 0.12 | 0.16 | 0.13 | 0.02 | 0.10 | 0.18 | 0.13 | 0.01 | 0.11 | 0.16 |
| CRLB PFC  glucose (mM) | 0.19 | 0.03 | 0.07 | 0.22 | 0.20 | 0.02 | 0.16 | 0.23 | 0.19 | 0.03 | 0.00 | 0.22 |
| CRLB PFC  glutamate (mM) | 0.17 | 0.04 | 0.10 | 0.22 | 0.17 | 0.05 | 0.09 | 0.22 | 0.17 | 0.04 | 0.09 | 0.22 |
| CRLB PFC  glutamine (mM) | 0.14 | 0.01 | 0.12 | 0.17 | 0.14 | 0.02 | 0.11 | 0.19 | 0.13 | 0.02 | 0.10 | 0.17 |
| CRLB PFC  glutathione (mM) | 0.07 | 0.01 | 0.06 | 0.09 | 0.07 | 0.01 | 0.06 | 0.10 | 0.08 | 0.01 | 0.06 | 0.09 |
| CRLB PFC macro-  molecules (arb.) | 10x  10^-4^ | 3x  10^-4^ | 7x  10^-4^ | 16x  10^-4^ | 11x  10^-4^ | 3x  10^-4^ | 7x  10^-4^ | 16x  10^-4^ | 10x  10^-4^ | 4x  10^-4^ | 7x  10^-4^ | 21x  10^-4^ |
| CRLB PFC  *myo*-inositol (mM) | 0.15 | 0.01 | 0.12 | 0.18 | 0.15 | 0.02 | 0.12 | 0.19 | 0.15 | 0.02 | 0.12 | 0.19 |
| CRLB PFC  NAA (mM) | 0.14 | 0.05 | 0.10 | 0.22 | 0.14 | 0.05 | 0.09 | 0.22 | 0.15 | 0.05 | 0.09 | 0.21 |
| CRLB PFC  NAAG (mM) | 0.09 | 0.01 | 0.07 | 0.11 | 0.09 | 0.01 | 0.06 | 0.13 | 0.09 | 0.01 | 0.07 | 0.12 |
| CRLB PFC  phosphoryl-ethanolamine (mM) | 0.17 | 0.01 | 0.16 | 0.20 | 0.17 | 0.02 | 0.13 | 0.24 | 0.17 | 0.02 | 0.14 | 0.22 |
| CRLB PFC  *scyllo*-inositol (mM) | 0.04 | 3x  10^-3^ | 0.03 | 0.04 | 0.04 | 4x  10^-3^ | 0.03 | 0.05 | 0.03 | 6x  10^-3^ | 0.00 | 0.04 |
| CRLB PFC  taurine (mM) | 0.15 | 0.01 | 0.13 | 0.17 | 0.14 | 0.01 | 0.12 | 0.18 | 0.14 | 0.02 | 0.11 | 0.18 |

Supplemental Table 7. Statistical results for follow-up analyses with co-variates included. Analyses were performed separately for each of the 6 metabolites examined *a priori*, in both OCC and PFC. Linear mixed-effects (LME) results show group differences in metabolite levels with age, sex assigned at birth, and body mass index (BMI) included as co-variates. SE = standard error. Significant effects (*p* < 0.05 uncorrected) are highlighted in bold. Non-significant trends (*p* < 0.1 uncorrected) are marked in italics.

| **Metabolite** | **LME with co-variates**  Controls vs. relatives | **LME with co-variates** Controls vs. PwPP | **LME with co-variates** Relatives vs. PwPP |
| --- | --- | --- | --- |
| OCC GABA | parameter estimate [SE] = 0.006 [0.04],  *t*_75_ = 0.15, *p* = 0.9 | parameter estimate [SE] = -0.02 [0.04],  *t*_124_ = -0.52, *p* = 0.6 | parameter estimate [SE] = -0.01 [0.05],  *t*_110_ = -0.12, *p* = 0.9 |
| OCC glutamate | parameter estimate [SE] = 0.01 [0.10],  *t*_75_ = 0.12, *p* = 0.9 | *parameter estimate [SE] = -0.18 [0.09],*  *t_124_ = -1.95, p = 0.054* | *parameter estimate [SE] = -0.17 [0.10],*  *t_110_ = -1.77, p = 0.079* |
| OCC glutamine | parameter estimate [SE] = -0.05 [0.08],  *t*_75_ = -0.66, *p* = 0.5 | parameter estimate [SE] = 0.07 [0.10],  *t*_124_ = 0.68, *p* = 0.5 | parameter estimate [SE] = 0.12 [0.10],  *t*_110_ = 1.14, *p* = 0.3 |
| OCC glutathione | parameter estimate [SE] = -0.03 [0.02],  *t*_75_ = -1.10, *p* = 0.3 | parameter estimate [SE] = -0.002 [0.02],  *t*_124_ = -0.11, *p* = 0.9 | parameter estimate [SE] = 0.01 [0.02],  *t*_110_ = 0.42, *p* = 0.7 |
| OCC NAA | parameter estimate [SE] = -0.18 [0.14],  *t*_75_ = -1.25, *p* = 0.2 | **parameter estimate [SE] = -0.25 [0.10],**  ***t*_124_ = -2.37, *p* = 0.019** | parameter estimate [SE] = -0.07 [0.12],  *t*_110_ = -0.60, *p* = 0.5 |
| OCC NAAG | parameter estimate [SE] = 0.004 [0.05],  *t*_75_ = 0.08, *p* = 0.9 | parameter estimate [SE] = -0.04 [0.04],  *t*_124_ = -1.0, *p* = 0.3 | parameter estimate [SE] = -0.05 [0.05],  *t*_110_ = -0.95, *p* = 0.3 |
| PFC GABA | parameter estimate [SE] = -0.09 [0.06],  *t*_45_ = -1.55, *p* = 0.13 | *parameter estimate [SE] = -0.10 [0.05],*  *t_87_ = -1.75, p = 0.083* | parameter estimate [SE] = -0.01 [0.05],  *t*_91_ = -0.11, *p* = 0.9 |
| PFC glutamate | parameter estimate [SE] = 0.002 [0.14],  *t*_45_ = 0.01, *p* = 1.0 | parameter estimate [SE] = -0.20 [0.15],  *t*_87_ = -1.32, *p* = 0.19 | parameter estimate [SE] = -0.22 [0.14],  *t*_91_ = -1.50, *p* = 0.14 |
| PFC glutamine | parameter estimate [SE] = -0.05 [0.09],  *t*_45_ = -0.57, *p* = 0.6 | parameter estimate [SE] = 0.06 [0.15],  *t*_87_ = 0.42, *p* = 0.7 | parameter estimate [SE] = 0.13 [0.13],  *t*_91_ = 0.97, *p* = 0.3 |
| PFC glutathione | parameter estimate [SE] = -0.06 [0.04],  *t*_45_ = -1.67, *p* = 0.10 | *parameter estimate [SE] = -0.05 [0.03],*  *t_87_ = -1.67, p = 0.098* | parameter estimate [SE] = 0.01 [0.03],  *t*_91_ = 0.37, *p* = 0.7 |
| PFC NAA | parameter estimate [SE] = -0.21 [0.16],  *t*_45_ = -1.33, *p* = 0.19 | *parameter estimate [SE] = -0.33 [0.17],*  *t*_87_ *= -1.97, p = 0.052* | parameter estimate [SE] = -0.15 [0.15],  *t*_91_ = -0.99, *p* = 0.3 |
| PFC NAAG | parameter estimate [SE] = -0.007 [0.04],  *t*_45_ = -0.15, *p* = 0.9 | parameter estimate [SE] = -0.06 [0.04],  *t*_87_ = -1.41, *p* = 0.16 | parameter estimate [SE] = -0.06 [0.04],  *t*_91_ = -1.45, *p* = 0.15 |

Supplemental Table 8. Statistical results for follow-up analyses within PwPP. Analyses were performed separately for each of the 6 metabolites examined *a priori*, in both OCC and PFC. Correlations are Spearman rank correlations. Significant effects (*p* < 0.05 uncorrected) are highlighted in bold.

| **Metabolite** | **Correlation with illness duration** PwPP only | **Correlation with CPZ equivalents** PwPP only |
| --- | --- | --- |
| OCC GABA | Spearman correlation, *r*_45_ =  -0.12, *p* = 0.4 | Spearman correlation, *r*_56_ =  0.10, *p* = 0.5 |
| OCC glutamate | Spearman correlation, *r*_45_ =  -0.18, *p* = 0.2 | Spearman correlation, *r*_56_ =  0.11, *p* = 0.4 |
| OCC glutamine | Spearman correlation, *r*_45_ =  0.15, *p* = 0.3 | Spearman correlation, *r*_56_ =  0.02, *p* = 0.9 |
| OCC glutathione | Spearman correlation, *r*_45_ =  0.15, *p* = 0.3 | Spearman correlation, *r*_56_ =  -0.04, *p* = 0.7 |
| OCC NAA | Spearman correlation, *r*_45_ =  -0.16, *p* = 0.3 | Spearman correlation, *r*_56_ =  0.09, *p* = 0.5 |
| OCC NAAG | **Spearman correlation, *r*_45_ =**  **0.32, *p* = 0.027** | Spearman correlation, *r*_56_ =  -0.04, *p* = 0.8 |
| PFC GABA | Spearman correlation, *r*_40_ =  -0.10, *p* = 0.5 | Spearman correlation, *r*_48_ =  0.05, *p* = 0.7 |
| PFC glutamate | Spearman correlation, *r*_40_ =  -0.16, *p* = 0.3 | Spearman correlation, *r*_48_ =  0.07, *p* = 0.6 |
| PFC glutamine | Spearman correlation, *r*_40_ =  0.11, *p* = 0.5 | Spearman correlation, *r*_48_ =  0.12, *p* = 0.4 |
| PFC glutathione | Spearman correlation, *r*_40_ =  0.09, *p* = 0.6 | Spearman correlation, *r*_48_ =  0.03, *p* = 0.8 |
| PFC NAA | Spearman correlation, *r*_40_ =  0.05, *p* = 0.8 | Spearman correlation, *r*_48_ =  0.11, *p* = 0.5 |
| PFC NAAG | **Spearman correlation, *r*_40_ =**  **0.35, *p* = 0.024** | Spearman correlation, *r*_48_ =  0.02, *p* = 0.9 |

Supplemental Table 9. Statistical results for follow-up analyses of glucose focused on effects of demographic factors. Analyses were performed separately for glucose in both OCC and PFC. Kruskal-Wallis (KW) results show group differences (males vs. females) in glucose levels across all participants. Correlations are Spearman rank correlations. Significant effects (*p* < 0.05 uncorrected) are highlighted in bold. Visualizations of the correlations between glucose and BMI are provided in Supplemental Figure 4.

| **Metabolite** | **KW sex**  all participants | **Correlation with age**  all participants | **Correlation with BMI**  all participants | **Correlation with illness duration** PwPP only | **Correlation with CPZ equivalents** PwPP only |
| --- | --- | --- | --- | --- | --- |
| OCC glucose | *X*^2^_2, n = 141_ = 0.005, *p* = 0.9 | Spearman correlation, *r*_139_ =  0.07, *p* = 0.4 | **Spearman correlation, *r*_120_ = 0.26, *p* = 0.004** | Spearman correlation, *r*_45_ =  0.16, *p* = 0.3 | Spearman correlation, *r*_56_ =  -0.14, *p* = 0.3 |
| PFC glucose | *X*^2^_2, n = 112_ = 0.09, *p* = 0.8 | Spearman correlation, *r*_110_ =  0.10, *p* = 0.3 | Spearman correlation, *r*_92_ =  0.13, *p* = 0.2 | Spearman correlation, *r*_40_ =  0.15, *p* = 0.3 | Spearman correlation, *r*_48_ =  -0.11, *p* = 0.4 |

Supplemental Figure 4. Correlations between body mass index (BMI) and glucose (Glc) levels. Data are shown for glucose levels in OCC (A) and PFC (B), units are millimolar (mM). The number of data points (n) and Spearman correlation (*r* and *p*) values are reported. Color indicates participant group: green = controls, blue = relatives, red = PwPP. Black line shows linear trend across all participants.

Supplemental Figure 5. OCC metabolites across controls (C), people with schizophrenia (SZ), and people with bipolar disorder (BP). Metabolites examined *a priori*: panels E, G-I, K; exploratory: panels A-D, I, J, L-N. Thick lines show group medians, boxes show 25-75%, whiskers show 1.5x interquartile range, dots show individual data points. Metabolite abbreviations not defined in the main text are as follows: glutamate (Glu), glutamine (Gln), glutathione (GSH), ascorbic acid (Asc), aspartic acid (Asp), total choline (tCho), total creatine (tCr), glucose (Glc), macromolecules (MacM), *myo*-inositol (mIns), phosphorylethanolamine (PE), *scyllo*-inositol (sIns), taurine (Tau). For our exploratory analyses, we applied false discovery rate (FDR) correction for multiple comparisons. Stars and lines indicate significant differences between 2 groups at *p* < 0.05, plus at *p* < 0.1.

Supplemental Figure 6. PFC metabolites across controls (C), people with schizophrenia (SZ), and people with bipolar disorder (BP). Metabolites examined *a priori*: panels E, G-I, K; exploratory: panels A-D, I, J, L-N. Thick lines show group medians, boxes show 25-75%, whiskers show 1.5x interquartile range, dots show individual data points. Metabolite abbreviations not defined in the main text are as follows: glutamate (Glu), glutamine (Gln), glutathione (GSH), ascorbic acid (Asc), aspartic acid (Asp), total choline (tCho), total creatine (tCr), glucose (Glc), macromolecules (MacM), *myo*-inositol (mIns), phosphorylethanolamine (PE), *scyllo*-inositol (sIns), taurine (Tau). For our exploratory analyses, we applied false discovery rate (FDR) correction for multiple comparisons. Stars and lines indicate significant differences between 2 groups at *p* < 0.05, plus at *p* < 0.1.

Supplemental Table 10. Metabolite concentrations for sub-groups of PwPP. Data are reported as means and standard deviations (SD) for occipital (OCC) and prefrontal (PFC) VOIs in participants with schizophrenia (SZ; n = 29 in OCC, n = 23 in PFC), and bipolar disorder (BP; n = 20 in OCC and PFC). Units are millimolar (mM), except for macromolecules (arbitrary units).

| **Metabolite** | **SZ, mean** | **SZ, SD** | **BP, mean** | **BP, SD** |
| --- | --- | --- | --- | --- |
| OCC ascorbic acid | 1.62 | 0.18 | 1.59 | 0.21 |
| OCC aspartic acid | 1.17 | 0.44 | 1.29 | 0.42 |
| OCC total choline | 1.04 | 0.14 | 1.02 | 0.11 |
| OCC total creatine | 8.54 | 0.51 | 8.16 | 0.34 |
| OCC GABA | 1.07 | 0.20 | 1.08 | 0.20 |
| OCC glucose | 1.26 | 0.47 | 1.26 | 0.63 |
| OCC glutamate | 8.49 | 0.45 | 8.50 | 0.38 |
| OCC glutamine | 2.82 | 0.47 | 2.94 | 0.51 |
| OCC glutathione | 0.87 | 0.12 | 0.81 | 0.09 |
| OCC macromolecules | 0.077 | 0.004 | 0.076 | 0.003 |
| OCC *myo*-inositol | 7.25 | 0.92 | 6.54 | 0.90 |
| OCC NAA | 10.86 | 0.48 | 10.75 | 0.49 |
| OCC NAAG | 1.77 | 0.24 | 1.69 | 0.24 |
| OCC phosphorylethanolamine | 1.13 | 0.21 | 1.07 | 0.21 |
| OCC *scyllo*-inositol | 0.25 | 0.13 | 0.26 | 0.12 |
| OCC taurine | 1.61 | 0.30 | 1.67 | 0.27 |
| PFC ascorbic acid | 1.52 | 0.22 | 1.60 | 0.17 |
| PFC aspartic acid | 1.54 | 0.53 | 1.76 | 0.31 |
| PFC total choline | 1.68 | 0.23 | 1.68 | 0.22 |
| PFC total creatine | 8.63 | 0.69 | 8.62 | 0.55 |
| PFC glucose | 1.09 | 0.25 | 1.13 | 0.28 |
| PFC GABA | 1.11 | 0.55 | 1.22 | 0.63 |
| PFC glutamate | 9.83 | 0.62 | 10.16 | 0.64 |
| PFC glutamine | 3.52 | 0.55 | 3.78 | 0.68 |
| PFC glutathione | 1.21 | 0.13 | 1.19 | 0.11 |
| PFC macromolecules | 0.069 | 0.007 | 0.072 | 0.007 |
| PFC *myo*-inositol | 7.59 | 0.90 | 7.16 | 0.83 |
| PFC NAA | 9.98 | 0.64 | 10.03 | 0.49 |
| PFC NAAG | 1.18 | 0.16 | 1.18 | 0.19 |
| PFC phosphorylethanolamine | 2.02 | 0.25 | 2.07 | 0.28 |
| PFC *scyllo*-inositol | 0.19 | 0.12 | 0.23 | 0.11 |
| PFC taurine | 1.88 | 0.33 | 2.07 | 0.28 |

Supplemental Table 11. Cramér-Rao lower bound (CRLB) values. Data (in absolute units [mM], not percentages) are reported as means, standard deviations (SD), minimum (min.) and maximum (max.) for occipital (OCC) and prefrontal (PFC) VOIs across participants with schizophrenia (SZ), and bipolar disorder (BP).

| **Metabolite** | **SZ, mean** | **SZ, SD** | **SZ, min** | **SZ, max** | **BP, mean** | **BP, SD** | **BP, min** | **BP, max** |
| --- | --- | --- | --- | --- | --- | --- | --- | --- |
| CRLB OCC  ascorbic acid (mM) | 0.17 | 0.01 | 0.15 | 0.20 | 0.16 | 0.01 | 0.15 | 0.20 |
| CRLB OCC  aspartic acid (mM) | 0.24 | 0.02 | 0.21 | 0.29 | 0.22 | 0.05 | 0.00 | 0.28 |
| CRLB OCC total  choline (mM) | 0.13 | 0.03 | 0.04 | 0.16 | 0.13 | 0.01 | 0.12 | 0.16 |
| CRLB OCC total  creatine (mM) | 0.44 | 0.04 | 0.34 | 0.53 | 0.42 | 0.04 | 0.35 | 0.50 |
| CRLB OCC  GABA (mM) | 0.14 | 0.01 | 0.12 | 0.16 | 0.13 | 0.01 | 0.11 | 0.15 |
| CRLB OCC  glucose (mM) | 0.22 | 0.01 | 0.20 | 0.26 | 0.21 | 0.02 | 0.19 | 0.25 |
| CRLB OCC  glutamate (mM) | 0.17 | 0.01 | 0.16 | 0.19 | 0.17 | 0.01 | 0.15 | 0.18 |
| CRLB OCC  glutamine (mM) | 0.14 | 0.01 | 0.12 | 0.17 | 0.14 | 0.01 | 0.11 | 0.17 |
| CRLB OCC  glutathione (mM) | 0.08 | 0.01 | 0.07 | 0.09 | 0.07 | 0.01 | 0.07 | 0.09 |
| CRLB OCC macro-  molecules (arb.) | 12x  10^-4^ | 4x  10^-4^ | 7x  10^-4^ | 21x  10^-4^ | 10x  10^-4^ | 3x  10^-4^ | 7x  10^-4^ | 15x  10^-4^ |
| CRLB OCC  *myo*-inositol (mM) | 0.15 | 0.02 | 0.12 | 0.18 | 0.14 | 0.02 | 0.11 | 0.19 |
| CRLB OCC  NAA (mM) | 0.19 | 0.05 | 0.10 | 0.24 | 0.17 | 0.05 | 0.10 | 0.23 |
| CRLB OCC  NAAG (mM) | 0.11 | 0.01 | 0.10 | 0.13 | 0.10 | 0.01 | 0.09 | 0.12 |
| CRLB OCC  phosphoryl-ethanolamine (mM) | 0.18 | 0.02 | 0.16 | 0.22 | 0.18 | 0.01 | 0.16 | 0.20 |
| CRLB OCC  *scyllo*-inositol (mM) | 0.04 | 0.01 | 0.00 | 0.04 | 0.04 | 0.00 | 0.03 | 0.04 |
| CRLB OCC  taurine (mM) | 0.15 | 0.01 | 0.12 | 0.17 | 0.14 | 0.01 | 0.12 | 0.17 |
| CRLB PFC  ascorbic acid (mM) | 0.17 | 0.02 | 0.14 | 0.20 | 0.17 | 0.02 | 0.14 | 0.20 |
| CRLB PFC  aspartic acid (mM) | 0.23 | 0.02 | 0.19 | 0.28 | 0.22 | 0.02 | 0.19 | 0.28 |
| CRLB PFC total  choline (mM) | 0.11 | 0.04 | 0.04 | 0.16 | 0.12 | 0.03 | 0.04 | 0.16 |
| CRLB PFC total  creatine (mM) | 0.45 | 0.07 | 0.33 | 0.70 | 0.45 | 0.05 | 0.38 | 0.55 |
| CRLB PFC  GABA (mM) | 0.14 | 0.01 | 0.11 | 0.16 | 0.13 | 0.01 | 0.12 | 0.15 |
| CRLB PFC  glucose (mM) | 0.19 | 0.04 | 0.00 | 0.22 | 0.19 | 0.01 | 0.16 | 0.22 |
| CRLB PFC  glutamate (mM) | 0.19 | 0.03 | 0.11 | 0.22 | 0.16 | 0.05 | 0.09 | 0.21 |
| CRLB PFC  glutamine (mM) | 0.14 | 0.02 | 0.11 | 0.17 | 0.14 | 0.02 | 0.11 | 0.16 |
| CRLB PFC  glutathione (mM) | 0.08 | 0.01 | 0.06 | 0.09 | 0.07 | 0.01 | 0.06 | 0.09 |
| CRLB PFC macro-  molecules (arb.) | 11x  10^-4^ | 4x  10^-4^ | 7x  10^-4^ | 21x  10^-4^ | 9x  10^-4^ | 3x  10^-4^ | 7x  10^-4^ | 15x  10^-4^ |
| CRLB PFC  *myo*-inositol (mM) | 0.15 | 0.02 | 0.12 | 0.19 | 0.14 | 0.02 | 0.12 | 0.17 |
| CRLB PFC  NAA (mM) | 0.16 | 0.05 | 0.09 | 0.21 | 0.14 | 0.05 | 0.09 | 0.20 |
| CRLB PFC  NAAG (mM) | 0.09 | 0.01 | 0.07 | 0.12 | 0.09 | 0.01 | 0.07 | 0.12 |
| CRLB PFC  phosphoryl-ethanolamine (mM) | 0.18 | 0.02 | 0.14 | 0.21 | 0.17 | 0.02 | 0.15 | 0.22 |
| CRLB PFC  *scyllo*-inositol (mM) | 0.03 | 0.01 | 0.00 | 0.04 | 0.03 | 0.00 | 0.03 | 0.04 |
| CRLB PFC  taurine (mM) | 0.15 | 0.02 | 0.11 | 0.18 | 0.14 | 0.02 | 0.12 | 0.17 |

Supplemental Figure 7. OCC hierarchical clustering dendrogram. The height of each bar indicates the distance between the clustered variables joined by that link. Colors indicate which of the clusters (*k* = 6) each variable was included in (also see Figure 4). Variable names are listed in Supplemental Table 3. Note that OCC glucose (variable #19) is the only MRS variable in the red cluster, which includes clinical (e.g., BPRS, variable #7) and visual measures (e.g., contrast discrimination, variable #1).

Supplemental Figure 8. OCC hierarchical clustering results for PwPP only. Cells show pairwise Spearman correlation *r* values for measures from visual tasks (CSS discrimination & suppression, COP accuracy & jitter threshold, SFM switch rate [Hz] & coefficient of variance [CV]), clinical assessments (BPRS, BACS, SGI, SPQ, PID-5 psychoticism, visual acuity, Mars contrast sensitivity), and MRS in OCC (Asc, Asp, tCr, GABA, Glc, Gln, Glu, tCho, GSH, mIns, MacM, NAA, NAAG, PE, sIns, Tau). See Supplemental Table 4 for a full list of variables. Colored dashed lines indicate the boundaries of various clusters (*k* = 4). Red text indicates glucose, which clustered with visual task and clinical measures (purple dashed box).

Supplemental Figure 9. OCC hierarchical clustering dendrogram for PwPP only. The height of each bar indicates the distance between the clustered variables joined by that link. Colors indicate which of the clusters (*k* = 4) each variable was included in (also see Supplemental Figure 8). Variable names are listed in Supplemental Table 3. OCC glucose (variable #19) clustered with psychiatric (e.g., BPRS, variable #7) and visual measures (e.g., contrast surround suppression, variable #2; purple cluster).

Supplemental Figure 10. OCC+PFC hierarchical clustering results. Cells show pairwise Spearman correlation *r* values for measures from visual tasks (CSS discrimination & suppression, COP accuracy & jitter threshold, SFM switch rate [Hz] & coefficient of variance [CV]), clinical assessments (BPRS, BACS, SGI, SPQ, PID-5 psychoticism, visual acuity, Mars contrast sensitivity), and MRS in OCC and PFC (Asc, Asp, tCr, GABA, Glc, Gln, Glu, tCho, GSH, mIns, MacM, NAA, NAAG, PE, sIns, Tau). See Supplemental Table 4 for a full list of variables. Colored dashed lines indicate the boundaries of various clusters (*k* = 7). Red text indicates glucose in OCC and PFC, which clustered with visual task and clinical measures, as well as NAAG in OCC and PFC (yellow box).

Supplemental Figure 11. OCC+PFC clustering dendrogram. The height of each bar indicates the distance between the clustered variables joined by that link. Colors indicate which of the clusters (*k* = 7) each variable was included in (see also Supplemental Figure 10). Variable names are listed in Supplemental Table 4. Glucose in OCC (variable #19) and PFC (#35) were clustered with measures of psychiatric and visual function (e.g., BPRS, variable #7; contrast discrimination, variable #1; yellow cluster).

Supplemental Figure 12. Scree plots from principal component analyses. Plots show the percentage of variance explained across principal components (black line) and number of clusters (*k*) chosen for each hierarchical clustering analysis. A) OCC clustering, data from all participants. B) OCC clustering, data from PwPP only. C) OCC+PFC clustering, data from all participants.

# Supplemental References

1	Demro C, Mueller BA, Kent JS, Burton PC, Olman CA, Schallmo M-P, et al. The psychosis human connectome project: An overview. NeuroImage. 2021;241:118439.

2	Schallmo M-P, Weldon KB, Kamath RS, Moser HR, Montoya SA, Killebrew KW, et al. The psychosis human connectome project: Design and rationale for studies of visual neurophysiology. NeuroImage. 2023;272:120060.

3	Jeste DV, Palmer BW, Appelbaum PS, Golshan S, Glorioso D, Dunn LB, et al. A new brief instrument for assessing decisional capacity for clinical research. Archives of general psychiatry. 2007;64(8):966-74.

4	Killebrew KW, Moser HR, Grant AN, Marjanska M, Sponheim SR, Schallmo M-P. Faster bi-stable visual switching in psychosis. Translational Psychiatry. 2024;14(201):1-11.

5	Montoya SA, Moser HR, Chong L-S, Grant AN, Sponheim SR, Engel SA, et al. Center-surround processing in psychosis. Biological Psychiatry: Cognitive Neuroscience and Neuroimaging. in press.

6	Tkáč I, Andersen P, Adriany G, Merkle H, Ugurbil K, Gruetter R. In Vivo 1H NMR Spectroscopy of the Human Brain at 7 T. Magnetic Resonance in Medicine. 2001;46:451-56.

7	Peirce JW. PsychoPy—Psychophysics software in Python. Journal of Neuroscience Methods. 2007;162(1-2):8-13.

8	Boynton GM, Demb JB, Glover GH, Heeger DJ. Neuronal basis of contrast discrimination. Vision Research. 1999;39:257-69.

9	Legge GE, Foley JM. Contrast masking in human vision. Journal of the Optical Society of America. 1980;70(12):1458-71.

10	Yu C, Klein SA, Levi DM. Cross-and iso-oriented surrounds modulate the contrast response function: The effect of surround contrast. Journal of Vision. 2003;3:527-40.

11	Zenger-Landolt B, Heeger DJ. Response suppression in V1 agrees with psychophysics of surround masking. The Journal of Neuroscience. 2003;23(17):6884-93.

12	Schallmo M-P, Grant AN, Burton PC, Olman CA. The effects of orientation and attention during surround suppression of small image features: A 7 Tesla fMRI study. Journal of Vision. 2016;16(10):19.

13	Kingdom FAA, Prins N. Psychophysics: A practical introduction*.* Academic Press: London; 2010.

14	Kamath RS, Weldon KB, Moser HR, Abdullahi KS, Burton PC, Sponheim SR, et al. Impaired contour object perception in psychosis. Biological Psychiatry: Cognitive Neuroscience and Neuroimaging. 2026;11(2):229-41.

15	Silverstein SM, Kovacs I, Corry R, Valone C. Perceptual organization, the disorganization syndrome, and context processing in chronic schizophrenia. Schizophrenia Research. 2000;43(1):11-20.

16	Silverstein S, Hatashita-Wong M, Schenkel L, Wilkniss S, Kovacs I, Feher A, et al. Reduced top-down influences in contour detection in schizophrenia. Cognitive Neuropsychiatry. 2006;11(2):112-32.

17	Silverstein S, Berten S, Essex B, Kovacs I, Susmaras T, Little DM. An fMRI examination of visual integration in schizophrenia. Journal of Integrative Neuroscience. 2009;8(2):175-202.

18	Silverstein SM, Harms MP, Carter CC, Gold JM, Keane BP, MacDonald A, et al. Cortical contributions to impaired contour integration in schizophrenia. Neuropsychologia. 2015;75:469-80.

19	Ullman S. The interpretation of structure from motion. Proceedings of the Royal Society B: Biological Sciences. 1979;203:405-26.

20	Treue S, Husain M, Andersen RA. Human perception of structure from motion. Vision Research. 1991;31(1):59-75.

21	Marjańska M, McCarten JR, Hodges J, Hemmy LS, Grant A, Deelchand DK, et al. Region-specific aging of the human brain as evidenced by neurochemical profiles measured noninvasively in the posterior cingulate cortex and the occipital lobe using 1H magnetic resonance spectroscopy at 7 T. Neuroscience. 2017;354:168-77.

22	Behar KL, Rothman DL, Spencer DD, Petroff C. Analysis of macromolecule resonances in 1H NMR spectra of human brain. Magnetic Resonance in Medicine. 1994;32:294-302.

23	Glasser MF, Sotiropoulos SN, Wilson JA, Coalson TS, Fischl B, Andersson JL, et al. The minimal preprocessing pipelines for the Human Connectome Project. NeuroImage. 2013;80:105-24.

24	Haijma SV, Van Haren N, Cahn W, Koolschijn PCMP, Hulshoff Pol HE, Kahn RS. Brain Volumes in Schizophrenia: A Meta-Analysis in Over 18 000 Subjects. Schizophrenia Bulletin. 2013;39(5):1129-38.

25	Brugger SP, Howes OD. Heterogeneity and Homogeneity of Regional Brain Structure in Schizophrenia: A Meta-analysis. JAMA Psychiatry. 2017;74(11):1104.

26	Fischl B. FreeSurfer. NeuroImage. 2012;62(2):774-81.

27	Leucht S, Samara M, Heres S, Davis JM. Dose Equivalents for Antipsychotic Drugs: The DDD Method: Table 1. Schizophrenia Bulletin. 2016;42(suppl 1):S90-S94.

28	Leucht S, Crippa A, Siafis S, Patel MX, Orsini N, Davis JM. Dose-Response Meta-Analysis of Antipsychotic Drugs for Acute Schizophrenia. American Journal of Psychiatry. 2020;177(4):342-53.

29	Yim O, Ramdeen KT. Hierarchical cluster analysis: Comparison of three linkage measures and application to psychological data. The Quantitative Methods for Psychology. 2015;11(1):8-21.

30	Petroff OAC, Yu RK, Ogino T. High-Resolution Proton Magnetic Resonance Analysis of Human Cerebrospinal Fluid. Journal of Neurochemistry. 1986;47(4):1270-76.

31	Bell JD, Brown JCC, Sadler PJ, MacLeod AF, Sönksen PH, Hughes RD, et al. High resolution proton nuclear magnetic resonance studies of human cerebrospinal fluid. Clinical Science. 1987;72(5):563-70.

32	Michaelis T, Merboldt K-D, Bruhn H, Hanicke W, Math D, Frahm J. Absolute concentrations of metabolites In the adult human brain in vivo: Quantification of localized proton MR spectra. Neuroradiology. 1993;187:219-27.
